# Analysis of subtelomeric virulence gene families in *Plasmodium falciparum* by comparative transcriptional profiling

**DOI:** 10.1111/j.1365-2958.2012.08019.x

**Published:** 2012-03-21

**Authors:** Kathrin Witmer, Christoph D Schmid, Nicolas M B Brancucci, Yen-Hoon Luah, Peter R Preiser, Zbynek Bozdech, Till S Voss

**Affiliations:** 1 Swiss Tropical and Public Health Institute4051 Basel, Switzerland; 2 University of Basel4003 Basel, Switzerland; 3 School of Biological Sciences, Nanyang Technological UniversitySingapore 639798

## Abstract

The *Plasmodium falciparum* genome is equipped with several subtelomeric gene families that are implicated in parasite virulence and immune evasion. Members of these families are uniformly positioned within heterochromatic domains and are thus subject to variegated expression. The best-studied example is that of the *var* family encoding the major parasite virulence factor *P. falciparum* erythrocyte membrane protein 1 (PfEMP1). PfEMP1 undergoes antigenic variation through switches in mutually exclusive *var* gene transcription. *var* promoters function as crucial regulatory elements in the underlying epigenetic control strategy. Here, we analysed promoters of upsA, upsB and upsC *var*, *rifA1*-type *rif*, *stevor*, *phist* and *pfmc-2tm* genes and investigated their role in endogenous gene transcription by comparative genome-wide expression profiling of transgenic parasite lines. We find that the three major *var* promoter types are functionally equal and play an essential role in singular gene choice. Unlike *var* promoters, promoters of non-*var* families are not silenced by default, and transcription of non-*var* families is not subject to the same mode of mutually exclusive transcription as has been observed for *var* genes. Our findings identified a differential logic in the regulation of *var* and other subtelomeric virulence gene families, which will have important implications for our understanding and future analyses of phenotypic variation in malaria parasites.

## Introduction

The blood stages of *Plasmodium falciparum* evade antibody-mediated host immunity by altering parasite-encoded antigens exposed on the surface of infected red blood cells (iRBCs) through antigenic variation (Biggs *et al*., 1991; Roberts *et al*., 1992; Smith *et al*., 1995; Kyes *et al*., 2001). Antigenic variation results from the amplification of hypervariable gene families, coupled to the ability to switch expression of individual genes. Notably, these gene families are predominantly located near the telomeres (Gardner *et al*., 2002). The positional clustering of antigen gene families in subtelomeric regions is not unique to *P. falciparum* but is common to a wide range of pathogenic protozoa and fungi (Myler *et al*., 1984; Barry *et al*., 2003; De Las *et al*., 2003; Keely *et al*., 2005; Deitsch *et al*., 2009). Subtelomeric gene families show high rates of recombination to facilitate diversity and this has been demonstrated in the model yeast *Schizosaccharomyces pombe* (Cooper *et al*., 1998) and in *P. falciparum*, where meiotic recombination among *var* genes is at least eight times more frequent than the estimated genomic average (Freitas-Junior *et al*., 2000; Taylor *et al*., 2000). Proximity to telomeres also modulates gene expression as exemplified by variegated silencing of genes nearby telomeres (Gottschling *et al*., 1990). Indeed, *P. falciparum* subtelomeric regions are heterochromatic and this repressive environment facilitates silencing and variegated gene expression (Flueck *et al*., 2009; Lopez-Rubio *et al*., 2009; Salcedo-Amaya *et al*., 2009).

Subtelomeric *P. falciparum* gene families include *var* (Baruch *et al*., 1995; Smith *et al*., 1995; Su *et al*., 1995), *rif* (repetitive interspersed family) (Weber, 1988), *stevor* (subtelomeric variable open reading frame) (Cheng *et al*., 1998), *phist* (*Plasmodium* helical interspersed subtelomeric) (Sargeant *et al*., 2006), *pfmc-2tm* (*P. falciparum* Maurer's clefts two transmembrane) (Sam-Yellowe *et al*., 2004) and the *hyp* families 1–17 (Sargeant *et al*., 2006). The best-studied example is that of the ∼ 60 member *var* gene family. *var* gene transcription is controlled by conserved promoter sequences that can be subgrouped into three main classes according to sequence and chromosomal location (Voss *et al*., 2000; Gardner *et al*., 2002; Kraemer and Smith, 2003; Lavstsen *et al*., 2003). PfEMP1, encoded by *var* genes, is exposed on the iRBC surface and mediates adherence to various endothelial receptors in the human host (Leech *et al*., 1984; Su *et al*., 1995; Kyes *et al*., 2001). The resulting sequestration of RBC aggregates in the microvasculature is a major cause of severe clinical outcomes such as cerebral and pregnancy-associated malaria (MacPherson *et al*., 1985; Carlson *et al*., 1990; Pongponratn *et al*., 1991; Rowe *et al*., 1995). Moreover, antigenic variation of PfEMP1 contributes substantially to chronic disease and transmission.

Unlike PfEMP1, the function of other exported protein families in parasite biology and disease is unclear, but there is indication for roles in host–parasite interactions (Fernandez *et al*., 1999; Kyes *et al*., 1999; Abdel-Latif *et al*., 2002; 2003; Lavazec *et al*., 2007; Schreiber *et al*., 2008). With over 130 members, the *rif* gene family is the largest in *P. falciparum* (Kyes *et al*., 1999; Joannin *et al*., 2008). A-type RIFINs are associated with parasite-induced membranous structures in the iRBC cytosol termed Maurer's clefts (MC) and the infected RBC surface. B-type RIFINs have been shown to reside within the parasite (Petter *et al*., 2007; 2008). Members of the ∼ 35-member STEVOR family localize to the surface of infected erythrocytes and are also associated with MCs (Lavazec *et al*., 2006; Blythe *et al*., 2008; Khattab *et al*., 2008; Niang *et al*., 2009). The PFMC-2TM family (12 members) are associated with MCs and the RBC surface (Sam-Yellowe *et al*., 2004; Lavazec *et al*., 2006). PHIST and HYP proteins are predicted to be exported to the iRBC (Sargeant *et al*., 2006), but this has not yet been experimentally confirmed.

*var* genes are transcribed in a mutually exclusive manner such that in single parasites only one *var* gene is active and all other members are silenced (Scherf *et al*., 1998). However, simultaneous transcription of two *var* loci has been observed in wild-type and transgenic parasites (Dzikowski *et al*., 2007; Brolin *et al*., 2009; Joergensen *et al*., 2010). *var* promoters are essential in establishing alternative states of activity (Dzikowski *et al*., 2006; 2007; Voss *et al*., 2006; 2007; Chookajorn *et al*., 2007; Lopez-Rubio *et al*., 2007). Transfection experiments using episomal promoters to drive expression of drug selectable markers proved a great strategy to investigate transcriptional regulation of *var* genes (Dzikowski *et al*., 2006; Voss *et al*., 2006; 2007). Upon transfection, *var* promoters are silenced by default (Calderwood *et al*., 2003; Frank *et al*., 2006; Voss *et al*., 2006; 2007) and this silenced state appeared to require the presence of the *var* gene intron (Deitsch *et al*., 2001; Frank *et al*., 2006). Subsequent studies showed that the intron is not strictly required for silencing; rather the *cis* pairing of a *var* promoter with the intron's own promoter activity, or that of other heterologous promoters, is important (Dzikowski *et al*., 2007). Interestingly, drug-induced selection for active promoters is sufficient to infiltrate drug-selectable markers into the pathway of mutually exclusive transcription (Dzikowski *et al*., 2006; Voss *et al*., 2006; 2007). In such parasites, the active episomal promoters cause a shutdown of endogenous *var* transcription and, consequently, absence of PfEMP1 on the iRBC surface. These studies have been instrumental not only in understanding the regulatory mechanisms underlying mutually exclusive transcription, but also in reconstructing earlier observations with regard to the variable phenotype of iRBCs (Voss *et al*., 2006; D'Ombrain *et al*., 2007; Elliott *et al*., 2007; Maier *et al*., 2008).

Despite most other multigene families in *P*. *falciparum* being known for many years, our understanding of the processes that regulate their transcription is limited. Expression of *rif*, *stevor* and *pfmc-2tm* genes is clonally variant *in vitro* and *in vivo* (Fernandez *et al*., 1999; Lavazec *et al*., 2007; Petter *et al*., 2007; 2008; Blythe *et al*., 2008; Mackinnon *et al*., 2009). Analysis of parasites selected for monogenic *var* expression provided no evidence for co-regulation between the active *var* and neighbouring *rif* or *stevor* genes (Sharp *et al*., 2006; Cabral and Wunderlich, 2009). These studies indicate that juxtaposition to active *var* genes does not predispose the activity of other gene family members. However, upsA and *rifA1* genes, a subset of *rif* genes positioned in head-to-head orientation with upsA *var* genes, were both upregulated in parasites selected to express PfEMP1 variants associated with severe disease in children (Wang *et al*., 2009). Moreover, a recent study observed interference of several episomal gene family promoters with transcription of endogenous gene families suggesting that multiple gene families may share a common transcriptional control mechanism (Howitt *et al*., 2009).

Here, we used genome-wide comparative transcriptional profiling to investigate the extent to which activation of extrachromosomal virulence gene promoters affects transcription of endogenous gene families. Our results establish a clear functional distinction between *var* and non-*var* promoters. They further demonstrate that *var* promoters of all types play an essential part in singular gene choice, and that transcription of non-*var* gene families is not subject to the same mode of transcriptional regulation that controls mutually exclusive transcription of *var* genes.

## Results

### Generation of stable reporter parasite lines

**Fig 1:**
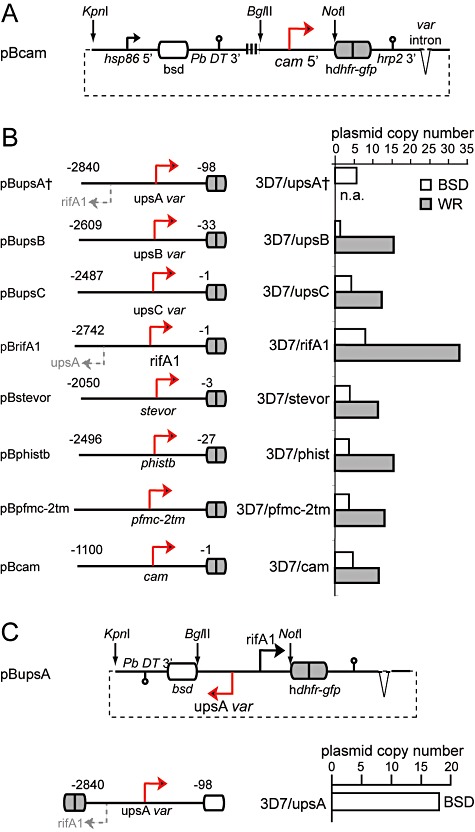
Transgenic reporter cell lines. A. Schematic map of the parental plasmid pBcam. The *bsd* resistance cassette (white box) selects for stable plasmid maintenance. The h*dhfr-gfp* cassette (grey box) allows selecting for active promoters of interest (red arrow). The *var* gene intron is indicated by a v-shape. A 0.5 kb TARE6 element is shown by vertical lines. hsp86 5′, *hsp86* promoter; *Pb* DT 3′, *P. berghei dhfr*-thymidilate synthase terminator; *hrp2* 3′, histidine-rich protein 2 terminator. B. Descendants of pBcam were obtained by replacing the *cam* promoter with promoters of interest (red arrows) using BglII and NotI. Numbers represent nucleotide positions in relation to the ATG start codon. Grey dashed arrows represent the bi-directional upsA/*rifA1* promoter. Plasmid names and cell lines are indicated. White and grey bars represent average plasmid copy numbers in unselected and WR-selected cell lines respectively. n.a., not available. C. Top: schematic map of pBupsA where the *bsd* and h*dhfr-gfp* selectable markers are controlled by the bi-directional upsA/*rifA1* promoter. Bottom: average plasmid copy number in BSD-selected 3D7/upsA parasites.

Parasites of the strain 3D7 were transfected with constructs carrying eight different promoters: upsA-type *var* (PF13_0003), upsB-type *var* (PFL0005w), upsC-type *var* (PFL1960w), *rifA1* (PF13_0004), *stevor* (PFL2610w), *phistb* (PFL2540w), *pfmc-2tm* (final gene ID unknown due to high sequence conservation between *pfmc-2tm* promoters) and *cam* (PF14_0323) (control). Note that the *rifA1* and upsA promoters are present on the sense and antisense strand, respectively, of the 2.8 kb intergenic sequence separating these head-to-head oriented genes. All sequences were cloned into the context of the parental reporter vector pBcam by replacement of the *cam* promoter (Fig. 1A). The blasticidin-S deaminase (*bsd*) resistance cassette was used to obtain transfectants carrying stable episomes. Importantly, this initial selection is independent of the activity of the test promoters driving transcription of the downstream drug-selectable reporter h*dhfr-gfp* (human dihydrofolate reductase fused to green fluorescent protein). All constructs carry a *var* gene intron element downstream of the h*dhfr-gfp* cassette for consistency. We chose this approach, rather than cloning family-specific introns, since the role of the *var* intron in silencing and mutual exclusion is exerted by the intron's own promoter activity (i.e. a functional feature of the intron) and is thus sequence-independent (Dzikowski *et al*., 2007).

The BSD-selected reporter lines 3D7/upsA†, 3D7/upsB, 3D7/upsC, 3D7/rifA1, 3D7/stevor, 3D7/phist, 3D7/pfmc-2tm and the control line 3D7/cam exhibited similar plasmid copy numbers ranging from two to eight copies per parasite (Fig. 1B). After WR99210 (WR) selection, average plasmid numbers increased with the highest number of 33 observed in 3D7/rifA1. Unexpectedly, 3D7/upsA† was completely refractory to WR selection in seven challenge experiments on three independently generated BSD-resistant populations. The reason for failure to select for active upsA promoters is unknown but may have been related to efficient upsA silencing and/or insufficient provision of stable h*dhfr* transcripts to confer WR resistance. In an attempt to circumvent these two possible obstacles, we exploited the possibility that *rifA1* promoter activity may predispose the head-to-head upsA promoter for activation. We therefore replaced the entire *bsd* cassette in pBrifA1 with an inverted *bsd* gene and terminator element to create pBupsA (Fig. 1C). In this context, the reverse complement sequence of the *rifA1* upstream region acts as an upsA promoter driving *bsd* transcription. Indeed, parasites transfected with this construct were successfully selected on BSD. Hence, this BSD-resistant population (3D7/upsA) carried an activated upsA promoter and was used in all subsequent experiments. Noteworthy, populations transfected with pBupsA were also successfully selected on WR, and reciprocal drug swaps had no effect on parasite growth suggesting that both activities of this bi-directional promoter were active simultaneously (data not shown). Southern blot analysis revealed that plasmids were maintained episomally in all lines except 3D7/stevor where integration into the endogenous *stevor* locus occurred (Fig. S1).

### Initial assessment of overall transcript profiles at four consecutive intervals during intra-erythrocytic development

**Fig 2:**
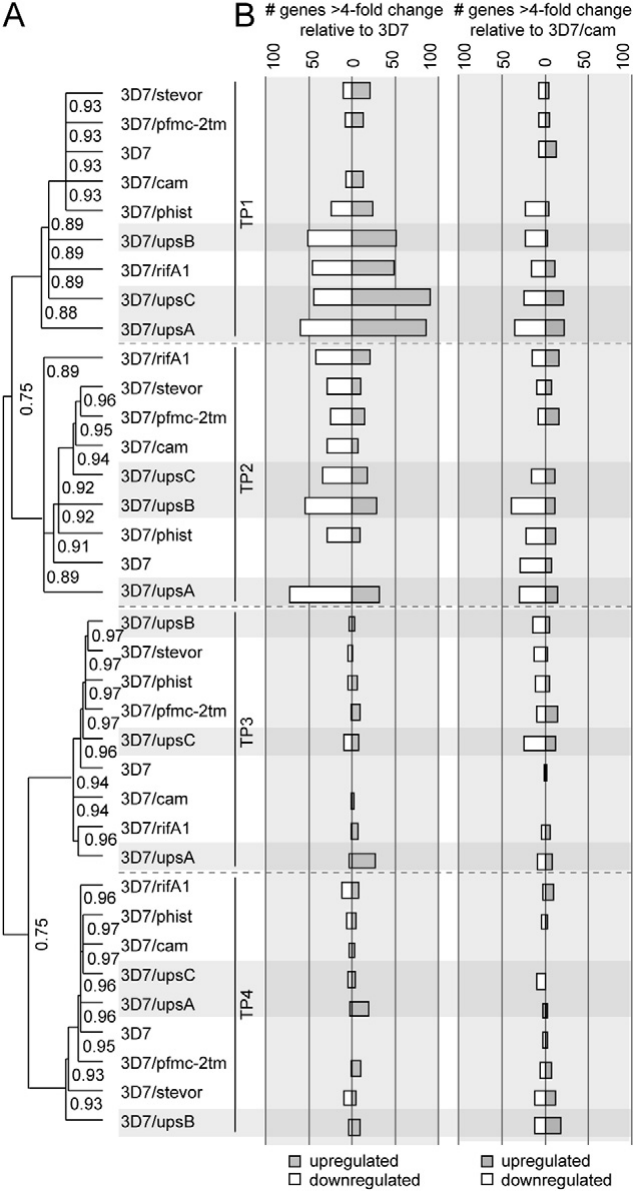
Hierarchical clustering of 36 transcriptomes. A. Hierarchical average linkage clustering of 3D7 and eight transgenic cell lines at four time points during the IDC. Numbers indicate similarity scores of two joined elements by uncentred Pearson correlation. TP, time point (TP1, 6–14 hpi; TP2, 14–22 hpi; TP3, 22–30 hpi; TP4, 30–38 hpi). B. Bars indicate the number of genes displaying more than fourfold change in relative expression (open bars, downregulated; grey bars, upregulated) in each cell line and time point relative to 3D7 wild type (left panel) or the 3D7/cam control transfectant (right panel). Parasites transfected with *var* promoter constructs are shaded grey.

We harvested total RNA at four consecutive time points [TP1: 6–14 h post invasion (hpi); TP2: 14–22 hpi; TP3: 22–30 hpi; TP4: 30–38 hpi] during the intra-erythrocytic developmental cycle (IDC) from 3D7 wild-type parasites and each of the WR-selected transfected lines (except for 3D7/upsA where a BSD-selected population was used). This sampling strategy was designed to include the temporal peaks of transcription for each endogenous gene family (Scherf *et al*., 1998; Kyes *et al*., 1999; 2000; Kaviratne *et al*., 2002; Sam-Yellowe *et al*., 2004). Relative transcript levels for each gene were determined relative to a 3D7 reference cDNA pool by hybridization to a genome-wide long oligonucleotide microarray (Hu *et al*., 2007) (GEO accession GSE31829). Hierarchical average linkage clustering revealed four distinct clusters corresponding to the four sampling time points (Fig. 2A). Within each time point, the samples showed a high degree of correlation but the individual datasets clustered differently. The overall similarity between individual transcriptomes was lowest in early ring stages and increased gradually with progression through the IDC (Fig. 2B). This is explained by the variegated expression of a large number of genes expressed specifically in ring-stage parasites (Bozdech *et al*., 2003; Marti *et al*., 2005; Llinas *et al*., 2006). Compared with both control parasites (3D7 wild type and 3D7/cam), the ring-stage transcriptomes of 3D7/upsA, 3D7/upsB, 3D7/upsC and 3D7/rifA1 displayed a higher number of deregulated genes than 3D7/stevor, 3D7/phist and 3D7/pfmc-2tm. Notably, all reporter line transcriptomes consistently showed a higher degree of similarity to the control transfectant 3D7/cam than to untransfected parasites (Fig. 2B). We explain this important observation by the likelihood that perturbations associated with the generation of transgenic parasites *per se* cause alterations in mRNA profiles. Indeed, we found 127 genes either up- or downregulated by more than twofold in at least one time point in all transfected lines compared with the untransfected control (Fig. S2). Interestingly, this set contained several genes coding for proteins involved in DNA/RNA metabolism, protein translation and export, and stress response. These changes are compatible with possible responses to plasmid maintenance and replication as well as to the mode of BSD action, which inhibits protein synthesis (Yamaguchi *et al*., 1965).

### Promoters of *var* and non-*var* subtelomeric gene families display distinct functional characteristics

As an important consideration for comparative analyses, we tested whether the stage-specific activities of the episomal promoters matched the temporal transcription profile of the cognate endogenous gene families. To do this, we compared reporter gene mRNA abundance profiles in selected lines to the average relative expression of the corresponding gene family members (Fig. S3). This analysis revealed high correlations for the *var*, *stevor*, *pfmc-2tm* and *cam* promoters demonstrating that the episomal sequences contained sufficient information to recapitulate the temporal expression of endogenous gene families. Intriguingly, the *rifA1* promoter correlated with endogenous *var* rather than *rif* transcription implying that this bi-directional *rif* promoter displays a *var*-specific activation pattern. This interesting observation may indicate differential regulation of this particular subset of *rif* genes compared with other members of the family. For the *phistb* promoter we observed divergence in early schizonts where episomal promoter activity increased and endogenous *phist* transcription decreased. The reason for this discrepancy is unclear but may be due to lack of the full complement of regulatory *cis*-acting sequences on the 2.5 kb upstream region cloned into pBphistb, or different mRNA stability properties of the reporter compared with endogenous *phist* transcripts.

**Fig 3:**
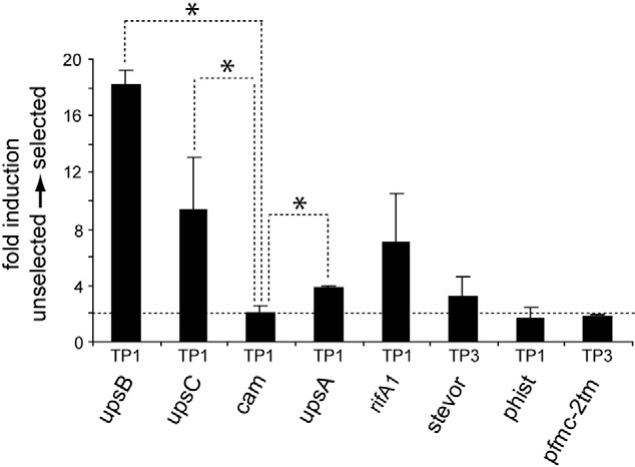
Gene family promoters other than *var* are not silenced by default. Relative reporter transcript levels were determined by qRT-PCR [normalized against transcription of arginyl-tRNA synthetase (PFL0900c) and adjusted for plasmid copy numbers]. The bars represent the average increase in relative promoter activity in drug-selected compared with unselected parasites at the time of peak transcription (fold increase). Values derive from three independent biological replicates (mean ± s.d.) (see also Fig. S4). Note that the value for the upsA promoter was calculated by dividing the relative *bsd* transcript levels in 3D7/upsA by the relative h*dhfr-gfp* levels in unselected 3D7/upsA†. The horizontal dashed line indicates the level of induction observed for the control promoter *cam*. Significant levels of induction compared with the negative control line 3D7/cam are indicated by asterisks (*P* < 0.05). TP, time point.

Several studies reported that upsB and upsC promoters are regulated by epigenetic mechanisms and adopt either a silenced (default) or an active (activated) state (Dzikowski *et al*., 2006; Frank *et al*., 2006; Voss *et al*., 2006; 2007). By quantifying steady-state mRNA levels in unselected (default) and drug-selected (activated) populations we asked if the promoters of upsA *var* and subtelomeric non-*var* families show the same behaviour. The *cam* promoter that is not epigenetically regulated and the upsB and upsC *var* promoters served as important negative and positive controls for silencing respectively. As expected, quantitative reverse transcription PCR (qRT-PCR) analysis revealed high levels of induction for the upsB promoter (18.2-fold ± 1.0 s.d.) and upsC promoter (9.4-fold ± 3.7 s.d.) relative to the negative control promoter *cam* (2.1-fold ± 0.4 s.d.) (Figs 3 and S4). Both the upsA (3.8-fold ± 0.6 s.d.) and *rifA1* promoters (7.1-fold ± 3.4 s.d.; not significant) displayed weaker but still elevated levels of induction compared with the control, which may be indicative for silencing of both activities of this bi-directional promoter in a considerable fraction of the unselected populations. In contrast, we found no evidence for alternative states of activity for the *stevor*, *phistb* and *pfmc-2tm* promoters. These results therefore suggest for the first time that promoters of subtelomeric non-*var* families may be unable to recruit transcriptional silencing autonomously. This is in distinction to *var* promoters that are characterized by alternative states of activity and reversible responsiveness to epigenetic control mechanisms (Deitsch *et al*., 2001; Duraisingh *et al*., 2005; Freitas-Junior *et al*., 2005; Voss *et al*., 2006; 2007; Chookajorn *et al*., 2007; Dzikowski *et al*., 2007; Lopez-Rubio *et al*., 2007).

### The capability to interfere with transcription of endogenous gene families is restricted to *var* gene promoters

**Fig 4:**
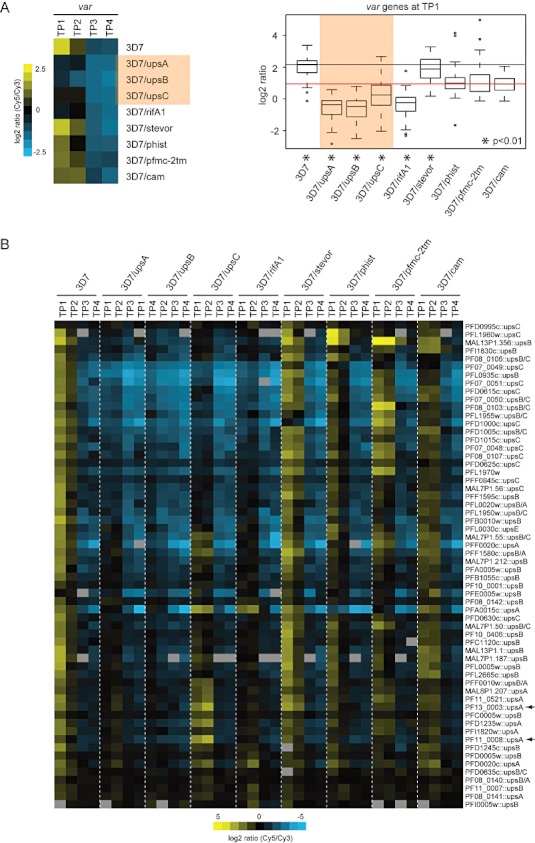
The capability to interfere with mutually exclusive *var* transcription is a specific feature of *var* gene promoters. A. Knock-down of endogenous *var* gene transcription. The heat-map reflects the mean relative expression of all *var* genes at four time points in each cell line (TP1–TP4). The box plot visualizes the distribution of relative *var* expression levels in all cell lines at the time of peak transcription (TP1). Parasites transfected with *var* promoter constructs are shaded. The box reflects the lower and upper quartiles, respectively, and the median is indicated by a horizontal line. Outliers are shown as dots. The solid red and black lines are drawn at the median for 3D7/cam and 3D7 respectively. Significant changes in relative *var* expression compared with the control transfectant 3D7/cam are indicated by asterisks (*P* < 0.01). B. The heat-map reflects relative transcript abundance for each *var* gene at all four time points (TP1–TP4) in all cell lines. Arrowheads identify two upsA *var* genes that were further analysed by qRT-PCR (Fig. S5).

Activation of upsB and upsC promoters leads to downregulation of endogenous *var* transcription (Dzikowski *et al*., 2006; 2007; Voss *et al*., 2006; 2007). Here, we studied the specificity of this cross-talk by comparative analysis of relative *var* transcription levels in all cell lines. As shown in Fig. 4A, mean relative *var* expression was strikingly reduced in early and late ring stages in 3D7/upsA, 3D7/upsB and 3D7/upsC compared with all other lines. Interestingly, this was also evident in 3D7/rifA1 to a level comparable to that induced by active *var* promoters. As expected, *var* transcription was repressed/absent in trophozoites and early schizonts (TP3 and TP4) in all lines investigated, underscoring the specificity of the changes observed for TP1 and TP2.

The box plot in Fig. 4A compares the distribution of relative *var* expression levels at TP1. With the exception of 3D7/stevor, average *var* transcription was reduced in all transfected lines compared with 3D7. Importantly, however, *var* downregulation was much less pronounced in 3D7/phist, 3D7/pfmc-2tm and 3D7/cam compared with the lines carrying activated *var* promoters. Compared with 3D7/cam, significant downregulation occurred specifically only in 3D7/upsA, 3D7/upsB, 3D7/upsC and 3D7/rifA1 parasites. This is also illustrated by comparing the relative expression levels for each individual *var* gene (Fig. 4B). In 3D7/upsA, 3D7/upsB and 3D7/rifA1 transcription of all *var* genes was largely reduced. In 3D7/upsC most *var* transcripts were also reduced; however, transcripts of the upsA and upsB/A *var* subgroups exhibited similar relative expression when compared with the 3D7 control. We would like to point out though that upsA and upsB/A genes are hardly expressed in unselected 3D7 parasites (Frank *et al*., 2007; Peters *et al*., 2007) and are expected only in minute amounts in the 3D7 cDNA reference pool. Consequently, the hybridization signals in 3D7/upsC most likely represent transcription slightly above background rather than significant mRNA levels. We addressed this possibility by quantifying two upsA transcripts by qRT-PCR and confirmed that both genes are hardly transcribed in 3D7/upsC as well as in the 3D7/cam control line (Fig. S5). However, we cannot exclude that *var* transcription is not always strictly mutually exclusive in all parasites in the 3D7/upsC population. Indeed, simultaneous expression of two *var* genes in a single parasite has been documented recently (Dzikowski *et al*., 2007; Brolin *et al*., 2009; Joergensen *et al*., 2010). Importantly, however, and in striking contrast to the *var* and *rifA1* promoter transfectants, transcripts of all *var* genes were detected in the 3D7/stevor, 3D7/phist, 3D7/pfmc-2tm and 3D7/cam populations. Compared with 3D7, the relative abundance of most transcripts was slightly lower and some were transcribed at higher levels in these lines.

**Fig 5:**
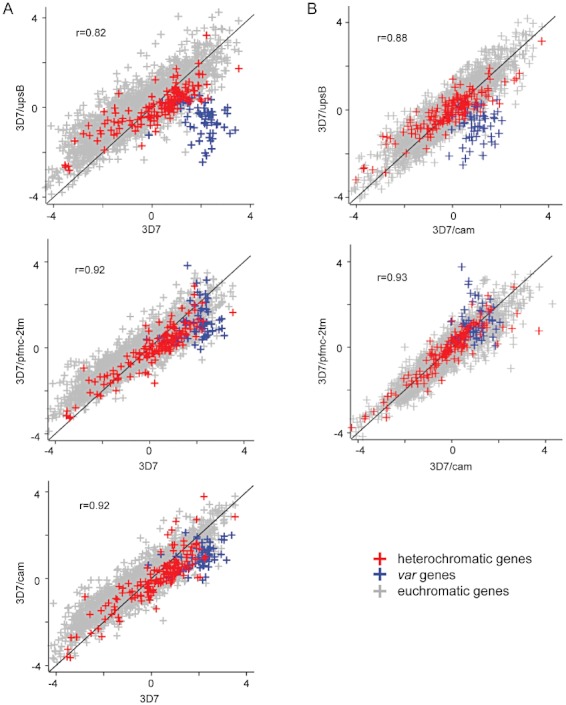
The scatter plots show the correlation of relative expression levels of all genes at TP1. Pearson correlation coefficients are indicated. *var* genes are highlighted in blue, heterochromatic genes in red (Flueck *et al*., 2009), all other genes are shown in grey. Values on the *y*- and *x*-axis represent log2 ratios for each gene in the two cell lines. A. 3D7/upsB (top), 3D7/pfmc-2tm (centre) and 3D7/cam (bottom) in comparison with 3D7 wild-type parasites. B. 3D7/upsB (top) and 3D7/pfmc-2tm (bottom) in comparison with the 3D7/cam control transfectant.

In addition to *var* genes, other heterochromatic genes also showed a trend towards downregulation in transfected versus untransfected parasites, irrespective of the promoter used to drive h*dhfr-gfp*. However, when comparing global transcription profiles to the control transfectant 3D7/cam this effect disappears except for *var* genes, which remain specifically downregulated (Fig. 5). We therefore conclude that transcription of heterochromatic genes is generally lower in episomally transfected parasites. This hypothesis is corroborated by the finding that 3D7/stevor, the only cell line where integration of the plasmid concatamer into the endogenous locus had occurred, was the only cell line displaying wild-type *var* transcript levels.

**Fig 6:**
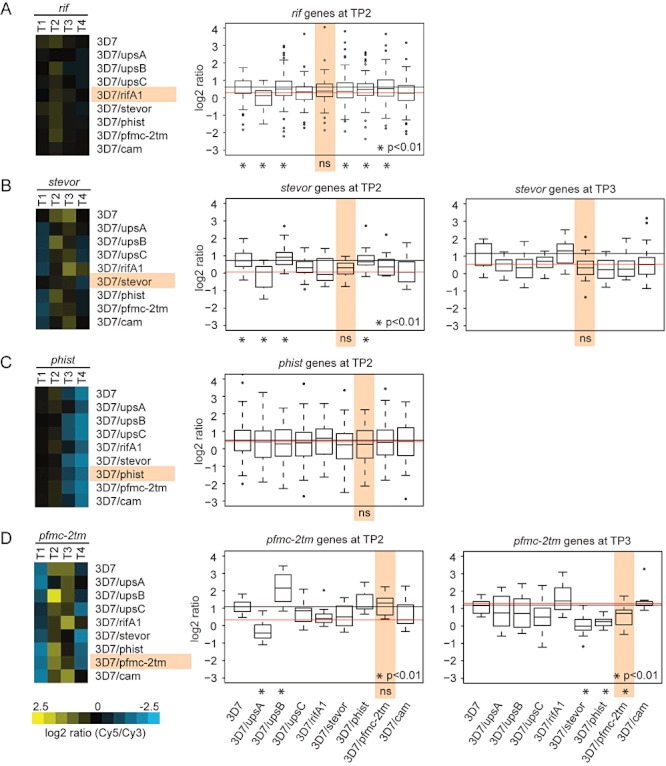
Multigene families other than *var* are not subject to promoter-induced mutual exclusion. Heat-maps reflect the mean relative expression of all members of the endogenous gene families at four time points in each cell line. Box plots visualize the distribution of relative expression levels for each gene family in all cell lines at the time of peak transcription. The box reflects the lower and upper quartiles, respectively, and the median is indicated by a horizontal line. Outliers are shown as dots. The solid red and black lines are drawn at the median for 3D7/cam and 3D7 respectively. Parasite lines transfected with the cognate promoter construct are shaded. Significant changes in relative expression compared with the control transfectant 3D7/cam are indicated by asterisks (*P* < 0.01). ns, not significant. A. Relative *rif* expression. B. Relative *stevor* expression. C. Relative *phist* expression. D. Relative *pfmc-2tm* expression.

Unlike the *var* promoters, neither of the *rifA1*, *stevor*, *phist* and *pfmc-2tm* promoters caused a specific downregulation in transcription of their cognate endogenous gene families (Fig. 6). We observed a trend towards reduced transcription of endogenous *stevor* and *pfmc-2tm* transcription in most transfected lines compared with wild-type 3D7, but this was also the case in the 3D7/cam control and hence not specific to any of the promoters tested. These data clearly demonstrate that transcriptional control of the *rif*, *stevor*, *phist* and *pfmc-2tm* family promoters does not obey the same strategy employed for *var* genes. These findings are in line with results from previous studies (Lavazec *et al*., 2007; Petter *et al*., 2007; Niang *et al*., 2009) underscoring the hypothesis that subtelomeric non-*var* families are not expressed by strict mutual exclusion.

In summary, we show that activation of the three main *var* promoter types, and a head-to-head *rifA1* promoter, results in silencing of endogenous *var* genes. Promoters of other gene families do not have this competence underscoring the specificity of *var* promoter-mediated interference with mutually exclusive *var* transcription. In contrast, variegated expression of other gene families occurs independently of promoter activation.

### Stochastic variation or true regulatory cross-talk?

Variegated expression of non-*var* families between individual lines was still obvious, and except for the *phist* families (Fig. 6C) we observed altered average expression in some transgenic lines relative to 3D7/cam. For instance, in late ring stages (TP2) *rif* and *stevor* transcription was slightly but significantly altered in several transfected lines (Fig. 6A and B). Average *pfmc-2tm* transcription was significantly reduced in 3D7/pfmc-2tm trophozoites but also in 3D7/stevor and 3D7/phist, showing that this downregulation was not specific to the active *pfmc-2tm* promoter. Moreover, in late ring stages average *pfmc-2tm* transcription was significantly lower in 3D7/upsA and higher in 3D7/upsB. If some or all of these altered profiles are indeed directly linked to the activity of particular promoter types and/or shared regulatory factors, or if they are simply due to stochastic variation on the population level remains to be investigated using more targeted approaches.

Next, we were interested in asking if any of the recently described and uncharacterized *hyp* families (Sargeant *et al*., 2006) were differentially expressed in our transfected cell lines. While the temporal transcription profile of most *hyp* genes was similar in all lines, we observed a striking upregulation in *hyp4* transcription in all time points in 3D7/pfmc-2tm parasites compared with all other lines (Fig. S6). This is particularly interesting given that all *hyp4* paralogues are located in head-to-tail orientation directly upstream of a *pfmc-2tm* locus. These observations suggest that expression of both families is co-regulated and that their exported gene products may cooperate in the same functional pathway in the iRBC. As a first step towards testing this hypothesis we generated a double transgenic cell line simultaneously expressing C-terminally tagged PFMC-2TM-GFP and HYP4-CherryFP and demonstrate that indeed HYP4 colocalizes with PFMC-2TM to MCs (Fig. S6).

Lastly, we addressed the interesting question as to whether *var* gene activation involves a single regulatory pathway, or if distinct transcriptional regulators mediate subtype-specific activation of *var* genes. To do this, we interrogated our datasets to identify genes with differential expression in the 3D7/upsA, 3D7/upsB and 3D7/upsC transfectants. This comparative approach identified only minor transcriptional differences between these cell lines. We pinpointed a total of only 20 transcripts with greater than threefold changes in relative expression in TP1 and TP2 in any one compared with both other cell lines. Eight, three and nine genes were differentially expressed in 3D7/upsA, 3D7/upsB and 3D7/upsC respectively (Fig. S7). Nearly all of these changes affected heterochromatic genes that are expected to undergo variegated expression. Interestingly, one of the genes upregulated in 3D7/upsA encodes the ApiAP2 factor PfSIP2 that has been implicated in silencing of subtelomeric upsB-type *var* genes (Flueck *et al*., 2010). However, apart from this interesting association this preliminary analysis failed to detect a clear link between distinct signatures of potential regulatory factors and activation of different *var* promoter subtypes, suggesting their activation is carried out by a common regulatory pathway.

## Discussion

*var* upstream regions are the dominant functional elements involved in the three main pillars of *var* gene regulation – silencing, activation and mutually exclusive locus recognition (Calderwood *et al*., 2003; Voss *et al*., 2003; 2006; 2007; Gannoun-Zaki *et al*., 2005; Dzikowski *et al*., 2006; 2007; Frank *et al*., 2006; Chookajorn *et al*., 2007). Here, we were able to expand our knowledge on this complex control strategy onto a genome-wide level, and to incorporate the analysis of upsA and several additional non-*var* promoters. Our comparative functional analysis separates the *var* and non-*var* subtelomeric gene families into two groups based on a different logic of transcriptional regulation.

The three main *var* promoter types share several functional attributes including similar relative activities and similar temporal activation profiles restricted to ring stages. Furthermore, *var* promoters adopt two different states of activity; silenced or active. While this behaviour has been described previously for upsB and upsC promoters, our results suggest for the first time that upsA promoters are regulated similarly (Dzikowski *et al*., 2006; Voss *et al*., 2006; 2007; Muhle *et al*., 2009). The relatively low level of induction observed for the upsA promoter in comparison with upsB and upsC has to be interpreted carefully. First, for technical reasons upsA-driven transcripts in unselected (3D7/upsA†) and selected (3D7/upsA) parasites differ in their coding (h*dhfr-gfp* versus *bsd*) and 3′ UTR (*hrp2* versus PbDT terminator) sequences. These differences may cause differential regulation at the level of mRNA stability, which in turn would affect the abundance of steady-state transcripts. Second, it is impossible to control or assess the proportion of parasites carrying active promoters prior to selection; differences in these proportions are probably reflected in the different default activity levels in 3D7/upsA†, 3D7/upsB and 3D7/upsC, which were lowest for upsB and highest for upsC. These uncertainties notwithstanding, activation of episomal upsA promoters led to a specific downregulation of endogenous *var* transcription as previously reported for upsB and upsC. We therefore believe that although the active upsA promoter was placed in a different plasmid context it still functioned as a regulatory entity equivalent to upsB and upsC promoters. Hence, despite considerable sequence variation between different *var* promoter types mutually exclusive activation of all *var* genes appears to be governed by a common regulatory mechanism. This assumption is also supported by our failure to identify distinct signatures of transcripts coding for potential transcriptional regulators in 3D7/upsA, 3D7/upsB and 3D7/upsC parasites.

Our initial attempts to select for an active upsA promoter by WR selection were unsuccessful. Likewise, a previous study failed to obtain activated upsA promoters based on drug selection (Muhle *et al*., 2009). The fact that the relative h*dhfr-gfp* mRNA levels in unselected 3D7/upsA† parasites were higher than those in most other WR-selected lines (Fig. S4) demonstrates that the refractoriness of 3D7/upsA† to WR challenge was not due to irreversible silencing. One possibility is that the upsA 5′ untranslated region (5′ UTR) mediates inefficient translation, which in turn may result in insufficient hDHFR concentrations to support WR resistance. Interestingly, the 5′ UTR of *var2csa* has been implicated in translational repression (Mok *et al*., 2008; Amulic *et al*., 2009), and we recently made similar observations for the upsC 5′ UTR (unpublished). Alternatively, it is also plausible that the phenotypic background in our 3D7 bulk population is rather incompatible with expression of upsA genes. In unselected cultured lines upsA genes are preferably silenced and have faster switch off rates than upsB and upsC genes (Dahlback *et al*., 2007; Frank *et al*., 2007; Peters *et al*., 2007). It would therefore be interesting to test if parasites selected for expression of upsA variants provide an environment suitable for activation of the pBupsA† construct. Enrichment for upsA-expressing cultured parasites can be achieved by repeated selection with antisera directed against upsA-type PfEMP1 variants (Jensen *et al*., 2004; Wang *et al*., 2009). Similarly, upsA *var* subtypes are also frequently expressed in clinical isolates and are associated with severe disease (Kirchgatter and Portillo, 2002; Bull *et al*., 2005; Kyriacou *et al*., 2006; Rottmann *et al*., 2006; Peters *et al*., 2007). This example suggests that environmental cues are important in determining the predisposition or capacity to express certain classes of *var* genes. The differential role of silent information regulator 2A (PfSIR2A) and PfSIR2B in silencing of *var* gene subgroups and neighbouring upsA *var* and *rifA1* genes (Duraisingh *et al*., 2005; Tonkin *et al*., 2009; Merrick *et al*., 2010) may provide an example of how such signals could be translated into changes in *var* expression. Likewise, the observed increase in transcription of *pfsip2* in the 3D7/upsA population may indicate a role in modulating *var* transcription. For instance, increased nuclear levels of PfSIP2 may be required for enhanced silencing of upsB genes in upsA-expressing parasites.

Analysis of the head-to-head *rifA1* promoter delivered several lines of evidence suggesting co-regulation of *rifA1* and upsA *var* genes. First, the temporal activity profile of this *rif* promoter matches that of endogenous *var* rather than *rif* genes. Interestingly, a recent study also provided evidence for a similar transcription profile (albeit at lower temporal resolution) of neighbouring upsA *var* and *rifA1* genes (Wang *et al*., 2009). Second, both promoters display increased activity levels in selected compared with unselected parasites. Third, and most compelling, selection for *rifA1* promoter activation induced an endogenous *var* knock-down similar to that achieved by *var* promoters. Overall, these results imply that activation of this subset of *rif* genes may be carried out by the same mechanisms and transcription factors that are also required for mutually exclusive activation of upsA genes. Interestingly, head-to-head upsA and *rifA1* genes were also co-regulated in parasites selected with IgG antibodies from highly immune adults (Wang *et al*., 2009), and are similarly upregulated in absence of PfSIR2A (Duraisingh *et al*., 2005; Tonkin *et al*., 2009). In light of this apparent co-regulation *in vitro* and *in vivo* it will be interesting to test if upsA PfEMP1 and A1-type RIFINs also cooperate on the protein level. If the functional properties of the *rifA1* promoter analysed here is representative for the entire *rif* family remains to be investigated. Based on sequence features and subcellular localization patterns, the RIFIN family is grouped into several distinct subgroups with likely diverse functions, one of which consists of *rifA1* genes positioned head-to-head with upsA *var* genes (Petter *et al*., 2007; 2008; Joannin *et al*., 2008; 2011; Wang *et al*., 2009). It is therefore conceivable that functionally distinct *rif* gene subsets are controlled by alternative regulatory mechanisms. It this context, it has to be noted that high copy numbers of plasmids carrying a different *rif* promoter type also caused downregulation of *var* transcription (Howitt *et al*., 2009). However, due to differences in the experimental set-up of that study compared with ours it remains unknown whether this effect can be attributed to true interference with mutually exclusive *var* transcription (see below).

The *stevor*, *phistb* and *pfmc-2tm* promoters showed no evidence for reversible states of activity indicating that these promoters are not directly targeted by the silencing machinery. Both the *stevor* and *pfmc-2tm* promoters were properly controlled in terms of temporal activity during the IDC. The *phistb* promoter was active coincident with endogenous *phist* transcription in ring stages and trophozoites, but remained active in early schizonts when endogenous *phist* transcription was reduced. If this discrepancy is due to the absence of critical *cis*-acting elements on the 2.5 kb episomal promoter, or to differences in mRNA stability between the episomal and endogenous transcripts is unclear. However, in light of the substantial activity displayed by the *phistb* promoter we envisage it was still controlled by the same transcription factors that also mediate endogenous *phist* transcription in ring stages and trophozoites. Importantly, for all three promoters we observed no specific downregulation in transcription of the endogenous *rif*, *stevor*, *phistb* and *pfmc-2tm* families. In these parasites the corresponding plasmid-encoded and endogenous promoters were active simultaneously, rather than in a mutually exclusive manner. In summary, these results demonstrate that transcriptional regulation of non-*var* families is controlled by mechanisms different from those governing mutually exclusive *var* transcription. They furthermore suggest that transcription of non-*var* families is not mutually exclusive, which is consistent with previous reports showing that more than one RIFIN or STEVOR variant can be expressed concurrently in a single parasite (Lavazec *et al*., 2007; Petter *et al*., 2007; Niang *et al*., 2009).

In a recent study, Howitt and colleagues observed that in parasites carrying high copy numbers of unpaired *var*, *rif*, *stevor* or *pfmc-2tm* promoters transcription of endogenous *var*, *stevor* and *pfmc-2tm* genes was decreased compared with wild-type parasites and to parasites carrying low copy numbers (Howitt *et al*., 2009). This downregulation occurred in a family-transcending manner and to variable degrees depending on the promoters and gene families analysed. To explain these observations, the authors proposed the existence of a common titratable nuclear factor in activating subtelomeric gene families (Howitt *et al*., 2009). This hypothesis was based on several important results obtained by the same group in earlier studies. Episomal *var* promoters were only silenced and counted by the mutual exclusion system if paired with a second promoter such as the *var* intron or a heterologous promoter (Deitsch *et al*., 2001; Calderwood *et al*., 2003; Frank *et al*., 2006; Dzikowski *et al*., 2007). Yet, unpaired *var* promoters still caused downregulation of endogenous *var* transcription if present in high plasmid copy numbers, but this effect was attributed to absorption of a titratable activation factor rather than true infiltration of the episome into the mutual exclusion system (Dzikowski and Deitsch, 2008). Here, by using an improved experimental set-up (including a control transfectant, paired promoters, sampling of consecutive IDC time points and genome-wide analyses) we are now able to revisit the above hypothesis and to distinguish between specific and non-specific effects. Similarly to Howitt and colleagues, we observed reduced transcription of *var* genes and other subtelomeric gene families in all transfected cell lines. However, the only specific effect occurred in 3D7/upsA, 3D7/upsB, 3D7/upsC and 3D7/rifA1 where paired *var* and the *rifA1* promoters caused specific silencing of endogenous *var* transcription by interference with mutually exclusive transcription. In contrast, the moderate degree of *var* downregulation in the remaining transfectants, and the reduced transcription of *stevor* and *pfmc-2tm* genes, is a likely result of non-specific mechanisms related or identical to those observed using unpaired promoter constructs (Dzikowski and Deitsch, 2008; Howitt *et al*., 2009). Notably, this effect also occurred to the same extent in the control transfectant 3D7/cam. Hence, our results are inconsistent with absorbance of a limiting factor specifically by subtelomeric virulence gene promoters; they rather indicate that subtelomeric gene transcription is generally reduced in transfected parasites. At this stage we can only speculate about the nature of this non-specific effect. It is conceivable that high plasmid copy numbers would absorb limiting factors critical for the expression of heterochromatic genes such as histone variants, chromatin remodelers and/or general transcription factors. Alternatively, reduced transcription of non-essential genes in transfected populations may be a direct or indirect consequence of mechanisms that compensate for a possible reduction in parasite fitness due to drug selection and forced maintenance of large concatenated plasmids. The transcriptional changes commonly observed in all transfected lines may be a reflection of such compensatory mechanisms.

A currently accepted model proposes the existence of a unique and physically restricted perinuclear zone dedicated to the expression of a single *var* locus only (Duraisingh *et al*., 2005; Ralph *et al*., 2005; Voss *et al*., 2006; Dzikowski *et al*., 2007; Lopez-Rubio *et al*., 2009). According to this concept, transcriptional *var* switching would occur through competition between a silenced and the active *var* gene. Our results are concordant with this hypothesis. Among the gene families investigated here, the mechanism by which singular *var* gene choice occurs appears to be unique and specific to the *var* gene family; promoters of other gene families do not interfere with this system. We envisage that specific *cis*-acting elements in *var* upstream regions could target *var* loci to such an expression site and that default silencing may be a prerequisite to protect *var* loci from illegitimate expression elsewhere in the nucleus. In fact, the reversible responsiveness of *var* promoters to silencing and activation may represent an inherent contributor to the overall mechanism controlling mutually exclusive transcription. On the contrary, *rif*, *stevor*, *phistb* and *pfmc-2tm* promoter activity is not mutually exclusive and appears to be mediated by an existing pool of readily available specific transcription factors. Recent genome-wide ChIP experiments demonstrated the ubiquitous enrichment of H3K9me3 and PfHP1 over *P. falciparum*-specific gene family islands throughout the genome (Flueck *et al*., 2009; Lopez-Rubio *et al*., 2009; Salcedo-Amaya *et al*., 2009). *var* gene activation is linked to the local exchange of H3K9me3/PfHP1 with H3K9ac and H3K4me2/3 predominantly in the upstream region (Lopez-Rubio *et al*., 2007). Such a functional epigenetic footprint is probably also involved in transcriptional activation of silenced non-*var* family members. However, the apparent lack of autonomous *cis*-acting elements capable of recruiting the silencing machinery to the *stevor*, *pfmc-2tm* and *phistb* promoters suggests that their expression may be passively controlled by *cis* and/or *trans* effects of neighbouring sequences that actively participate in structuring the local chromatin environment.

Overall, our study demonstrates a lack of systematic regulatory cross-talk between different subtelomeric gene families in *P. falciparum*. The most interesting circumstantial evidence for potential co-regulation is related to the *pfmc-2tm* family. First, we observed a significant down- and upregulation of *pfmc-2tm* genes in 3D7/upsA and 3D7/upsB respectively. It is worth mentioning that similar to our *in vitro* data a previous study reported inordinate *pfmc-2tm* expression patterns *in vivo* (Mackinnon *et al*., 2009). However, if *pfmc-2tm* expression is truly co-regulated with subtype-specific *var* gene transcription, or if these variations result from stochastic fluctuations in temporal *pfmc-2tm* activity, needs to be tested in future studies. Second, *hyp4* genes displayed a striking upregulation in 3D7/pfmc-2tm compared with all other cell lines. This result, together with the strict positional link between both gene types in the genome, and the colocalization of PFMC-2TM and HYP4 to MCs suggests that these two gene families may be functionally inter-connected.

To our knowledge, this is the first study comparing multiple transfected *P. falciparum* lines by global transcriptional profiling. As an important notion for future studies, we advise to use mock transfectants rather than untransfected parasites as controls for comparative transcriptome analyses involving transgenic lines. Another important technical aspect relates to the use of transgenic *P. falciparum* to study the function of variant surface antigens. While the ability to create PfEMP1 knock-down parasites using such a transfection-based approach continues to be informative for the analysis of immune responses to and binding properties of PfEMP1 (Voss *et al*., 2006; D'Ombrain *et al*., 2007; Elliott *et al*., 2007; Maier *et al*., 2008), this approach may not be feasible to achieve a complete knock-down of other suspected surface antigens and virulence factors.

In this work we shed important light on the transcriptional control of virulence gene families in *P. falciparum* and possibly that of other pathogens. We show that activation of the three major types of *var* gene promoters is equally important in the process of singular *var* gene choice in *P. falciparum*. The transition of a *var* gene promoter from the silenced to the activated state most likely represents an integral part of the overall mutual exclusion mechanism. We also demonstrate that this mode of regulation is unique to *var* genes; non-*var* families such as *rif*, *stevor*, *phist* and *pfmc-2tm* do not obey the same principle of mutually exclusive transcription. This difference in the control of *var* versus non-*var* gene families underscores the high significance of antigenic variation of PfEMP1 in parasite survival and transmission.

## Experimental procedures

### Parasite culture and transfection

*Plasmodium falciparum* 3D7 parasites were cultured as described previously (Trager and Jenson, 1978). Transfections were performed as described (Voss *et al*., 2006) and parasites were selected on 2.5 µg ml^−1^ BSD and 4 nM WR99210. Generation of transfection constructs is explained in detail in *Supporting information*. Growth synchronization was achieved by repeated sorbitol lysis (Lambros and Vanderberg, 1979). To obtain an 8 h growth window pre-synchronized parasites were synchronized twice 16 h apart.

### Transcriptional profiling by microarray analysis

For microarray analysis, total RNA was harvested at four consecutive time points: 6–14 hpi, 14–22 hpi, 22–30 hpi and 30–38 hpi. A pool of total RNA isolated from synchronized 3D7 parasites at five consecutive time points (10 h growth window each) covering the complete intra-erythrocytic developmental cycle was used as reference sample. Total RNA was prepared by disrupting 1.5 ml of RBC pellet (> 3% parasitaemia) with 10 ml of TriReagent (Sigma-Aldrich) following manufacturer's instructions. cDNA was generated from total RNA, and the test and reference samples were labelled as previously described (Bozdech *et al*., 2003) and hybridized to a long oligonucleotide genome-wide microarray containing 10 166 probes (Hu *et al*., 2007). The raw microarray data representing mRNA abundance ratios between each sample and the reference pool were subjected to lowess slide normalization and background filtering as implemented by the Acuity 4.0 program (Molecular Devices). The Cy5/Cy3 log ratios of multiple probes per gene were averaged. Genes with detectable signals in less than 75% of all samples as well as tRNA and rRNA genes were excluded from the analysis. All microarray data are available at the GEO repository (Accession No. GSE31829).

### Data analysis

Genes and arrays were clustered by average linkage clustering using Gene Cluster 3.0 (Eisen *et al*., 1998). The array tree and heatmaps were generated using the Java Treeview program (Saldanha, 2004). The cut-off for changes in relative expression was fourfold for pairwise comparisons (Fig. 2), and threefold (Fig. S7) or twofold (Fig. S2) to identify genes differentially expressed in one versus several other cell lines. Boxplots and scatter plots were generated using the statistical package R (version 2.10.0). Paired and unpaired *t*-tests were used to assess statistical significance between means of log2 ratios obtained from microarray hybridizations and qRT-PCR triplicate values respectively.

### Genomic DNA extraction, RNA extraction and cDNA synthesis

Parasites were released from iRBCs in 0.15% saponin. Total RNA was isolated using TriReagent (Sigma-Aldrich) and further purified using the RNeasy Plus Mini Kit (Qiagen) for removal of gDNA. Residual gDNA was digested with TURBO DNA-*free*™ (Ambion). All samples were tested negative for contaminating gDNA by quantitative PCR (qPCR). RNA was reverse transcribed using the RETROscript Kit® (Ambion). gDNA was extracted from the same parasite samples by addition of 3% SDS/0.27 M Na-acetate to the parasite pellet followed by phenol/chloroform extraction and precipitation. gDNA was resuspended in ddH_2_O and diluted 1:100 for qPCR analysis.

### Quantitative real-time PCR

For determination of the temporal promoter activity profiles RNA and gDNA were harvested from the same cultures that were also used for microarray analysis. For analysis of promoter activities in unselected and drug-selected populations, and to assess upsA transcripts, parasites were synchronized as described above and gDNA and total RNA was harvested at 6–14 hpi (TP1) for 3D7/upsA, 3D7/upsB, 3D7/upsC, 3D7/rifA1, 3D7/phist and 3D7/cam, and at 22–30 hpi (TP3) for 3D7/stevor and 3D7/pfmc-2tm respectively. qRT-PCR experiments were performed on three independent biological samples. Plasmid copy numbers were determined by qPCR on gDNA and calculated by dividing the absolute copy numbers obtained for h*dhfr-gfp* by the value obtained for the PFL0900c (arginyl-tRNA synthetase) locus (Frank *et al*., 2006). All reactions were run in triplicate yielding virtually identical Ct values. Five serial 1:10 dilutions of 3D7 genomic or plasmid DNA were used as standard for absolute quantification. Relative transcript profiles were calculated by normalization against the housekeeping gene PFL0900c. Cycling conditions were: 50°C, 2 min; 95°C, 10 min, followed by 40 cycles of 95°C, 15 s; 58°C, 1 min. Product-specific amplification was ensured by performing melting curves for each reaction. Reactions were performed at final primer concentrations of 0.4 µM using Power SYBR® Green Master Mix (Applied Biosystems) on a StepOnePlus™ Real-Time PCR System (Applied Biosystems) in a reaction volume of 12 µl. Primers are listed in Table S1.

### Live cell fluorescence microscopy

Five hundred microlitres of culture (5% haematocrit, 3–8% trophozoites) was pelleted for 1 min at 200 *g* and washed twice in 1 ml of RPMI-HEPES. The pellet was resuspended in 400 µl of RPMI-HEPES containing 2.5 µg ml^−1^ DAPI and incubated for 10 min at 37°C. Three microlitres of packed RBCs were mixed with 2 µl of Vectashield (Vector Laboratories) and immediately analysed on a Leica DM 5000B microscope. Images were taken at 100× magnification with a Leica DFC 300 FX camera and acquired via the Leica IM 1000 software, and processed using Adobe Photoshop CS2.

### Southern blotting

gDNA was subjected to Southern analysis using standard procedures. gDNA was digested with EcoRI/PvuII and separated on 0.7% agarose gels. Southern blots were probed with random-primed ^32^P-dATP-labelled h*dhfr*.
