# Supplementary material for: Analysis of subtelomeric virulence gene families in Plasmodium falciparum by comparative transcriptional profiling
Source: Mol Microbiol. 2012 Mar 21;84(2):243–59. doi: 10.1111/j.1365-2958.2012.08019.x (PMC3491689; doi:10.1111/j.1365-2958.2012.08019.x)
Supplement: Supplementary file 1 [file mmi0084-0243-SD1.pdf]

Supporting Information for:

Analysis of subtelomeric virulence gene families in *Plasmodium falciparum* by comparative transcriptional profiling

Kathrin Witmer[1,2], Christoph D. Schmid[1,2], Nicolas M. B. Brancucci[1,2], Yen-Hoon Luah[3], Peter R. Preiser[3], Zbynek Bozdech[3] and Till S. Voss[1,2,*]

[1] Swiss Tropical and Public Health Institute, 4051 Basel, Switzerland

[2] University of Basel, 4003 Basel, Switzerland

[3] School of Biological Sciences, Nanyang Technological University, Singapore 639798.

*For correspondence: E-mail till.voss@unibas.ch; Tel. +41 61 284 81 61; Fax +41 61 284 81 01

Contents:

- Supporting Experimental Procedures
- Supporting Figures and Legends (Figs. S1 to S7)
- Supporting Table 1

Supporting Experimental Procedures

All promoter constructs generated in this study are derivatives of pBcam which was itself obtained by the following procedure. The h*dhfr* gene in pBcam_MCS (Flueck *et al.*, 2009) was replaced with a h*dhfr-gfp* fusion gene. The h*dhfr-gfp* fusion was generated by ligation of a h*dhfr* PCR product into *BamH*I/*Sal*I-digested pGEM3-Zf(+) (Promega). The *Not*I site at the 5′ end of the forward primer is followed by a 5bp AAAACA sequence, which naturally occurs directly upstream of the PFL1960w *var* ATG. The *gfp* gene was amplified from pARLmTGFP (Struck *et al.*, 2005) and ligated in frame with the h*dhfr* gene into *Sal*I/*Avr*II-digested pGEM-h*dhfr*. The fusion gene was excised with *BamH*I/*Avr*II and cloned into *BamH*I/*Avr*II-digested pBcam_MCS to obtain pBcamHG. The *var* gene intron was amplified from pHBupsCRI (Voss *et al.*, 2006), digested with *Mfe*I and cloned into *EcoR*I-digested pBcamHG to obtain pBcam. 5′ upstream regions of interest were amplified by PCR from 3D7 gDNA, digested with *Bgl*II/*Not*I and cloned upstream of the h*dhfr-gfp* reporter gene into *Bgl*II/*Not*I-digested pBcam. The resulting constructs were named pBupsA†, pBupsB, pBupsC, pBrifA1, pBstevor, pBphistb and pBpfmc-2tm. To create plasmid pBupsA, the *bsd* gene and PbDT3′ terminator sequence was amplified from pBcam, digested with *Bgl*II/*Kpn*I and cloned in reverse orientation into *Bgl*II/*Kpn*I-digested pBrifA1

To generate pBcamHYP4-3xHA-CherryFP the CherryFP coding sequence was amplified from pCherry (kind gift from Paul Gilson, Burnet Institute, Melbourne, Australia) and cloned into *Sac*I/*Sal*I-digested pBcam-3xHA (Flueck *et al.*, 2009) to obtain pBcam-3xHA-CherryFP. A *hyp4* gene (gene ID unknown due to 99% sequence identity between paralogs) was amplified from 3D7 gDNA and cloned into *BamH*I/*Nhe*I-digested pBcam-3xHA-CherryFP. pHcamPFMC-2TM-GFP was obtained by replacing the *bsd* gene in pBcam-3xHA with the h*dhfr* gene amplified from pHBcam^{R} (Voss *et al.*, 2006) using *Xho*I. The 3xHA tag was then excised with *Not*I/*Sal*I and replaced with the *gfp* gene to obtain pHcam-GFP. The *pfmc-2tm* gene encoded by PFF0060w was amplified from 3D7 gDNA and inserted upstream of the *gfp* sequence using *BamH*I/*Not*I. Primer sequences are shown in Table S1.

Supporting Figures, Tables and Datasets

Figure S1

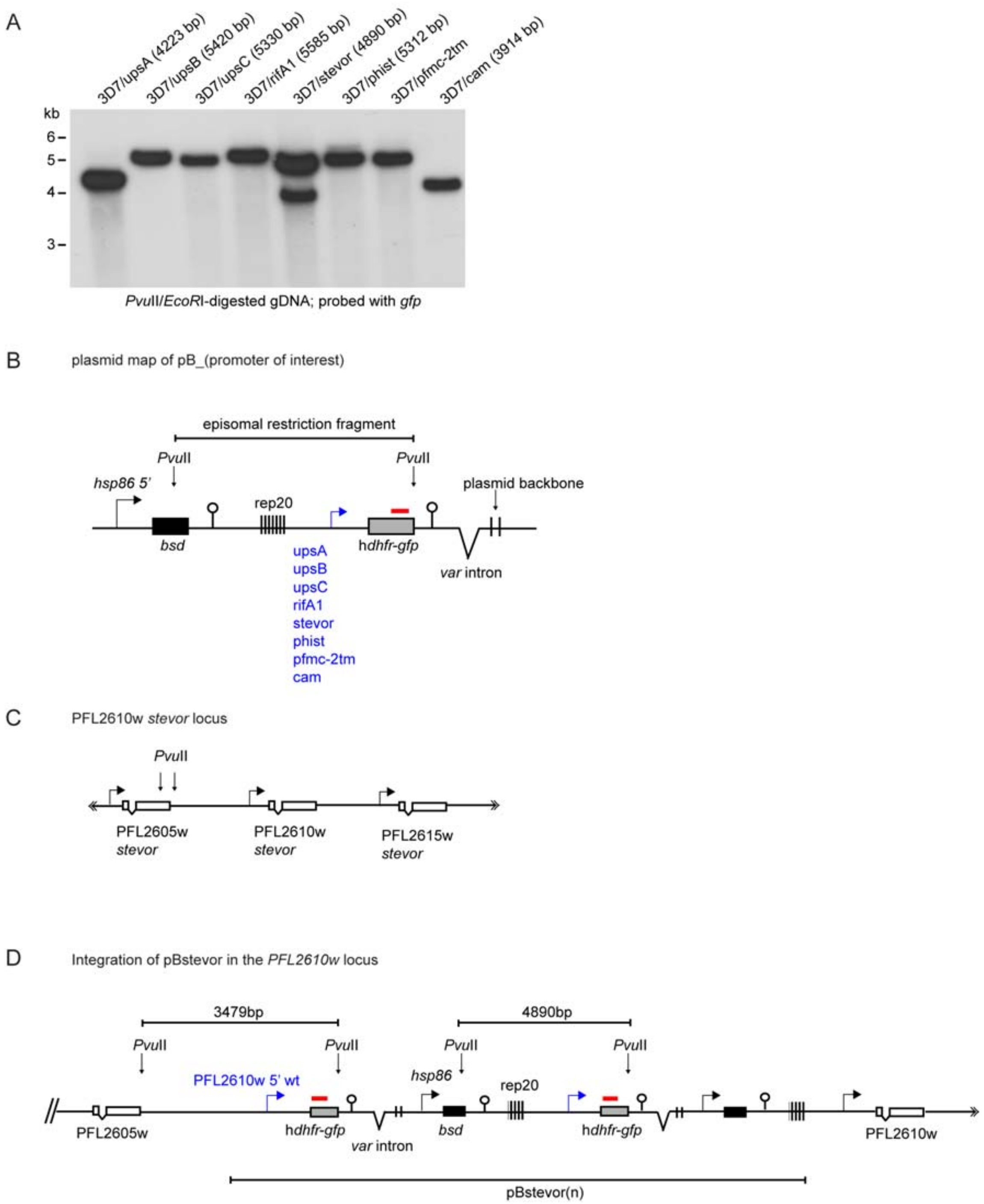

Fig. S1. Episomal and integrated plasmids.

A. Southern blot of gDNA isolated from drug-selected transgenic cell lines. gDNA was digested with *PvuII*/*Eco*RI. h*dhfr* was used as a probe to visualize episomal

fragments or plasmid integration into endogenous loci. The size of the resulting fragments is indicated for each cell line. The second fragment in 3D7/stevor reflects integration of pBstevor into the PFL2610w locus (see D).

B. Plasmid map of pBcam and derivatives.

C. Schematic map of the PFL2610w *stevor* locus.

D. Integration of pBstevor at the *stevor* locus PFL2610w. The position of *Pvu*II restriction sites and the size of the expected fragments hybridising with the h*dhfr* probe are highlighted.

Figure S2

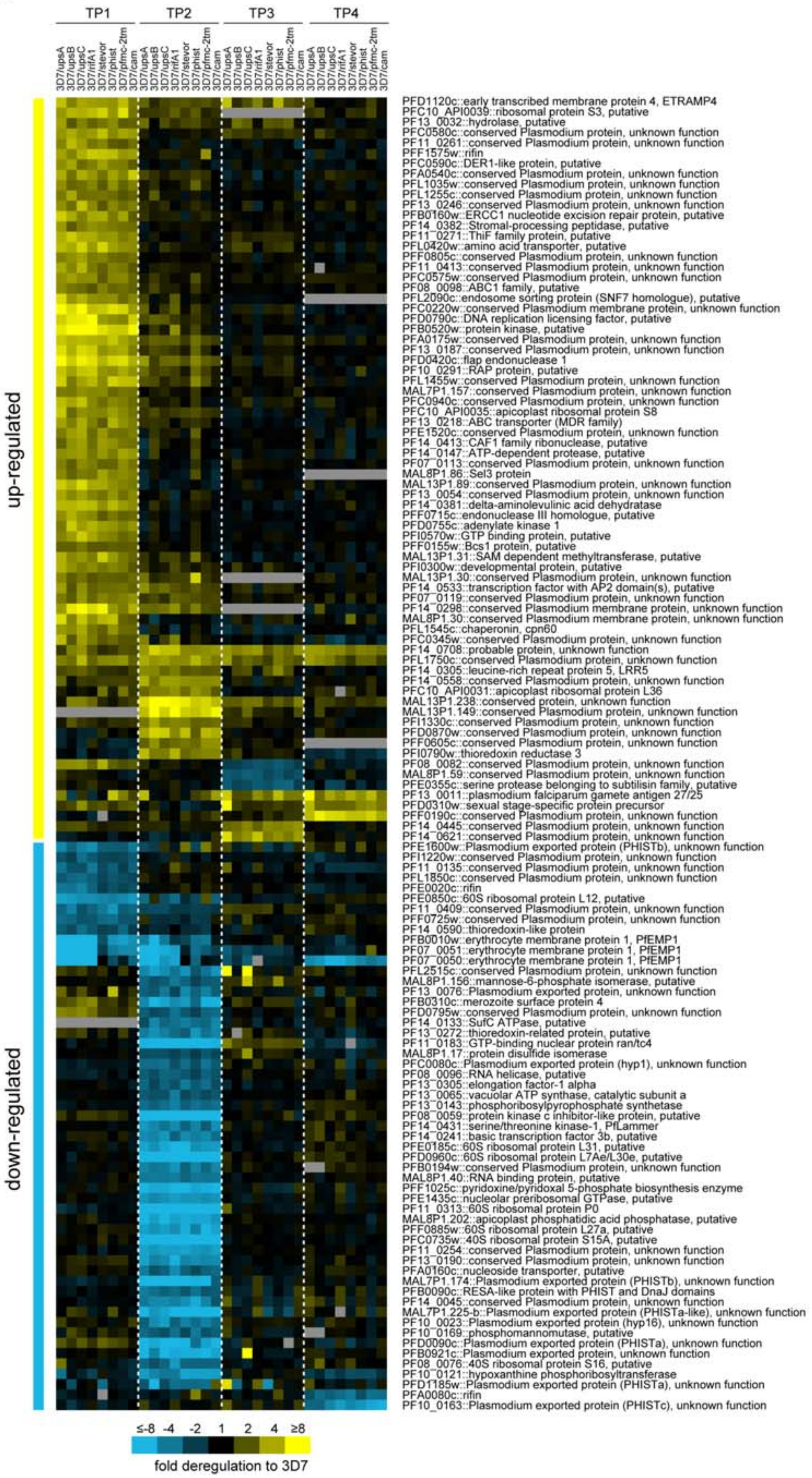
TP1
TP2
TP3
TP4
3D7/upsA
3D7/upsB
3D7/upsC
3D7/rifA1
3D7/stevor
3D7/phist
3D7/pfmc-2tm
3D7/cam
up-regulated
down-regulated
PFD1120c::early transcribed membrane protein 4, ETRAMP4
PFC10_API0039::ribosomal protein S3, putative
PF13_0032::hydrolase, putative
PFC0580c::conserved Plasmodium protein, unknown function
PF11_0261::conserved Plasmodium protein, unknown function
PFF1575w::rifin
PFC0590c::DER1-like protein, putative
PFA0540c::conserved Plasmodium protein, unknown function
PFL1035w::conserved Plasmodium protein, unknown function
PFL1255c::conserved Plasmodium protein, unknown function
PF13_0246::conserved Plasmodium protein, unknown function
PFB0160w::ERCC1 nucleotide excision repair protein, putative
PF14_0382::Stromal-processing peptidase, putative
PF11_0271::ThiF family protein, putative
PFL0420w::amino acid transporter, putative
PFF0805c::conserved Plasmodium protein, unknown function
PF11_0413::conserved Plasmodium protein, unknown function
PFC0575w::conserved Plasmodium protein, unknown function
PF08_0098::ABC1 family, putative
PFL2090c::endosome sorting protein (SNF7 homologue), putative
PFC0220w::conserved Plasmodium membrane protein, unknown function
PFD0790c::DNA replication licensing factor, putative
PFB0520w::protein kinase, putative
PFA0175w::conserved Plasmodium protein, unknown function
PF13_0187::conserved Plasmodium protein, unknown function
PFD0420c::flap endonuclease 1
PF10_0291::RAP protein, putative
PFL1455w::conserved Plasmodium protein, unknown function
MAL7P1.157::conserved Plasmodium protein, unknown function
PFC0940c::conserved Plasmodium protein, unknown function
PFC10_API0035::apicoplast ribosomal protein S8
PF13_0218::ABC transporter (MDR family)
PFE1520c::conserved Plasmodium protein, unknown function
PF14_0413::CAF1 family ribonuclease, putative
PF14_0147::ATP-dependent protease, putative
PF07_0113::conserved Plasmodium protein, unknown function
MAL8P1.86::Sel3 protein
MAL13P1.89::conserved Plasmodium protein, unknown function
PF13_0054::conserved Plasmodium protein, unknown function
PF14_0381::delta-aminolevulinic acid dehydratase
PFF0715c::endonuclease III homologue, putative
PFD0755c::adenylate kinase 1
PFI0570w::GTP binding protein, putative
PFF0155w::Bcs1 protein, putative
MAL13P1.31::SAM dependent methyltransferase, putative
PFI0300w::developmental protein, putative
MAL13P1.30::conserved Plasmodium protein, unknown function
PF14_0533::transcription factor with AP2 domain(s), putative
PF07_0119::conserved Plasmodium protein, unknown function
PF14_0298::conserved Plasmodium membrane protein, unknown function
MAL8P1.30::conserved Plasmodium membrane protein, unknown function
PFL1545c::chaperonin, cpn60
PFC0345w::conserved Plasmodium protein, unknown function
PF14_0708::probable protein, unknown function
PFL1750c::conserved Plasmodium protein, unknown function
PF14_0305::leucine-rich repeat protein 5, LRR5
PF14_0558::conserved Plasmodium protein, unknown function
PFC10_API0031::apicoplast ribosomal protein L36
MAL13P1.238::conserved protein, unknown function
MAL13P1.149::conserved Plasmodium protein, unknown function
PFI1330c::conserved Plasmodium protein, unknown function
PFD0870w::conserved Plasmodium protein, unknown function
PFF0605c::conserved Plasmodium protein, unknown function
PFI0790w::thioredoxin reductase 3
PF08_0082::conserved Plasmodium protein, unknown function
MAL8P1.59::conserved Plasmodium protein, unknown function
PFE0355c::serine protease belonging to subtilisin family, putative
PF13_0011::plasmodium falciparum gamete antigen 27/25
PFD0310w::sexual stage-specific protein precursor
PFF0190c::conserved Plasmodium protein, unknown function
PF14_0445::conserved Plasmodium protein, unknown function
PF14_0621::conserved Plasmodium protein, unknown function
PFE1600w::Plasmodium exported protein (PHISTb), unknown function
PFI1220w::conserved Plasmodium protein, unknown function
PF11_0135::conserved Plasmodium protein, unknown function
PFL1850c::conserved Plasmodium protein, unknown function
PFE0020c::rifin
PFE0850c::60S ribosomal protein L12, putative
PF11_0409::conserved Plasmodium protein, unknown function
PFF0725w::conserved Plasmodium protein, unknown function
PF14_0590::thioredoxin-like protein
PFB0010w::erythrocyte membrane protein 1, PfEMP1
PF07_0051::erythrocyte membrane protein 1, PfEMP1
PF07_0050::erythrocyte membrane protein 1, PfEMP1
PFL2515c::conserved Plasmodium protein, unknown function
MAL8P1.156::mannose-6-phosphate isomerase, putative
PF13_0076::Plasmodium exported protein, unknown function
PFB0310c::merozoite surface protein 4
PFD0795w::conserved Plasmodium protein, unknown function
PF14_0133::SufC ATPase, putative
PF13_0272::thioredoxin-related protein, putative
PF11_0183::GTP-binding nuclear protein ran/tc4
MAL8P1.17::protein disulfide isomerase
PFC0080c::Plasmodium exported protein (hyp1), unknown function
PF08_0096::RNA helicase, putative
PF13_0305::elongation factor-1 alpha
PF13_0065::vacuolar ATP synthase, catalytic subunit a
PF13_0143::phosphoribosylpyrophosphate synthetase
PF08_0059::protein kinase c inhibitor-like protein, putative
PF14_0431::serine/threonine kinase-1, PfLammer
PF14_0241::basic transcription factor 3b, putative
PFE0185c::60S ribosomal protein L31, putative
PFD0960c::60S ribosomal protein L7Ae/L30e, putative
PFB0194w::conserved Plasmodium protein, unknown function
MAL8P1.40::RNA binding protein, putative
PFF1025c::pyridoxine/pyridoxal 5-phosphate biosynthesis enzyme
PFE1435c::nucleolar preribosomal GTPase, putative
PF11_0313::60S ribosomal protein P0
MAL8P1.202::apicoplast phosphatidic acid phosphatase, putative
PFF0885w::60S ribosomal protein L27a, putative
PFC0735w::40S ribosomal protein S15A, putative
PF11_0254::conserved Plasmodium protein, unknown function
PF13_0190::conserved Plasmodium protein, unknown function
PFA0160c::nucleoside transporter, putative
MAL7P1.174::Plasmodium exported protein (PHISTb), unknown function
PFB0090c::RESA-like protein with PHIST and DnaJ domains
PF14_0045::conserved Plasmodium protein, unknown function
MAL7P1.225-b::Plasmodium exported protein (PHISTa-like), unknown function
PF10_0023::Plasmodium exported protein (hyp16), unknown function
PF10_0169::phosphomannomutase, putative
PFD0090c::Plasmodium exported protein (PHISTa), unknown function
PFB0921c::Plasmodium exported protein, unknown function
PF08_0076::40S ribosomal protein S16, putative
PF10_0121::hypoxanthine phosphoribosyltransferase
PFD1185w::Plasmodium exported protein (PHISTa), unknown function
PFA0080c::rifin
PF10_0163::Plasmodium exported protein (PHISTc), unknown function
≤-8 -4 -2 1 2 4 ≥8
fold deregulation to 3D7

91

Fig. S2. Heat map showing all genes commonly de-regulated >2-fold in at least one time point in all transgenic cell lines compared to 3D7 wild-type parasites. The colour scale indicates fold changes in relative transcript abundance ($2^{\Delta \log 2\ ratios}$) between each of the transfected lines compared to 3D7 wild-type parasites. GeneIDs and annotations are according to PlasmoDB version6.3.

Figure S3

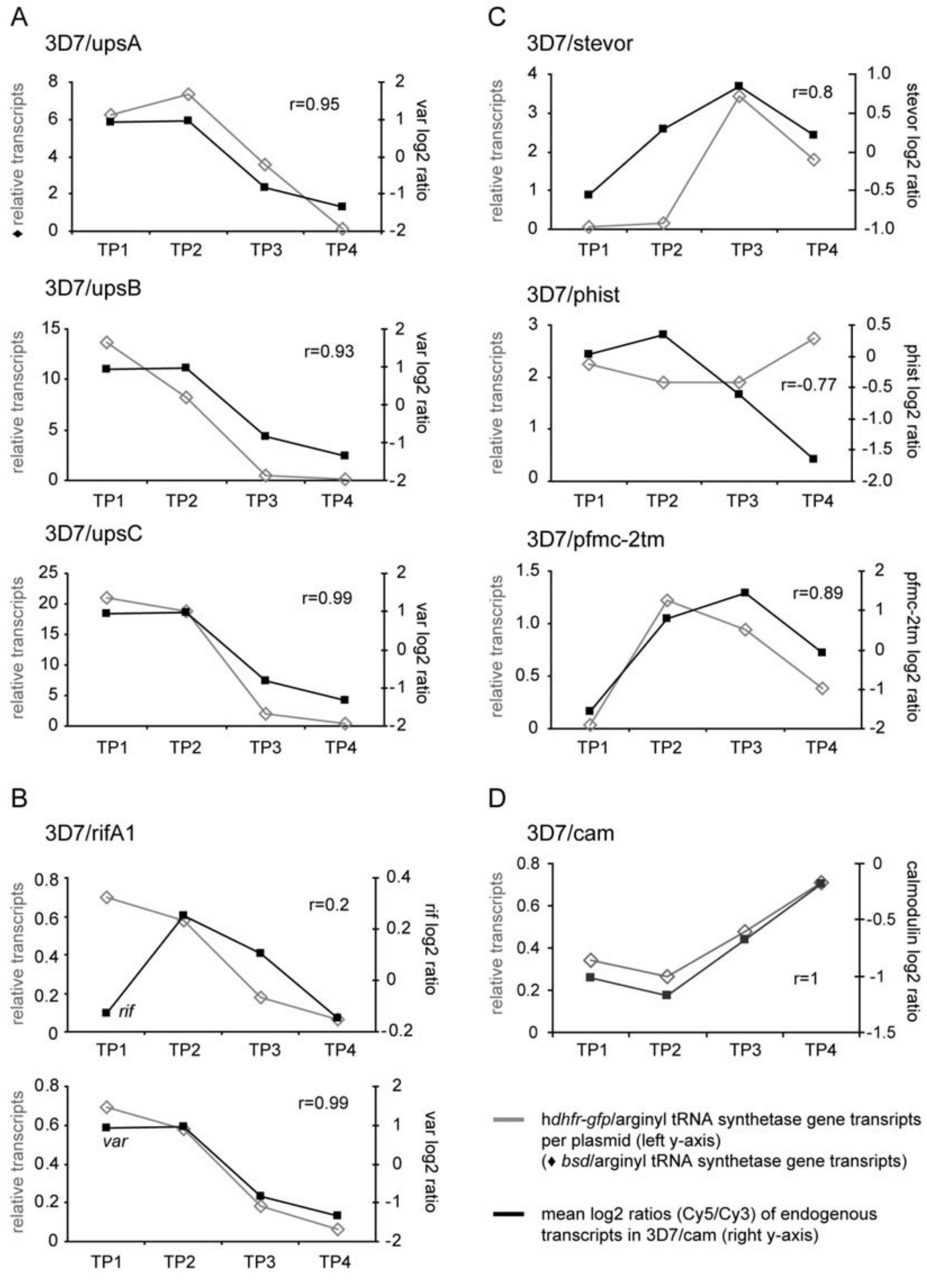

Fig. S3. Correlation between episomal promoter activity and endogenous gene transcription. Grey lines refer to the left axis and represent relative h*dhfr-gfp* mRNA levels produced per single promoter as determined by qRT-PCR. Values are

normalised against transcription of arginyl-tRNA Synthetase (PFL0900c) and adjusted for plasmid copy numbers. Black lines refer to the right axis and represent the mean relative expression of the cognate gene family members in 3D7/cam. Time points one to four (TP1 – TP4) are plotted on the x-axis. Pearson correlation coefficients are indicated.

A. Correlation between episomal upsA, upsB and upsC *var* promoter activities and endogenous *var* gene transcription. Note that upsA activity is reflected in relative *bsd* transcripts (black diamond).

B. Episomal *rifA1* promoter activity in correlation with endogenous *rif* (top) or *var* transcription (bottom).

C. Episomal *stevor, phistb* and *pfmc-2tm* activity in correlation with transcription of the corresponding endogenous gene families.

D. Correlation between relative activities of the episomal and endogenous *cam* promoters.

Figure S4

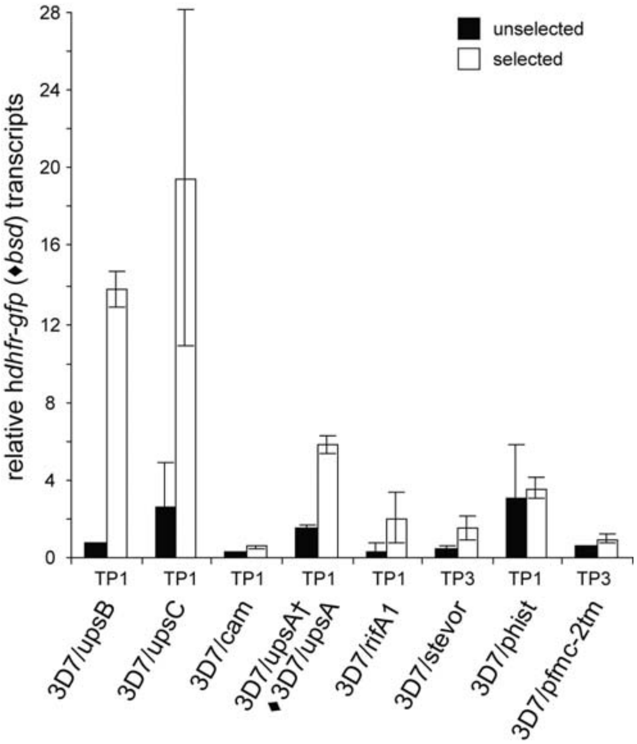

Fig. S4. Relative promoter activities in unselected (default) and drug-selected (activated) populations. Relative reporter transcript levels reflect episomal promoter activities in each transfectant before (black bars) and after (open bars) selection. Note that in 3D7/upsA the activated upsA promoter drives transcription of *bsd* (diamond) rather than h*dhfr-gfp*. Transcription of the house-keeping gene arginyl-tRNA synthetase (PFL0900c) was used for normalisation. Values have been adjusted for plasmid copy numbers. Total RNA was harvested three times independently at time points of peak activity. Values represent the mean +/- s.d. TP, time point.

Figure S5

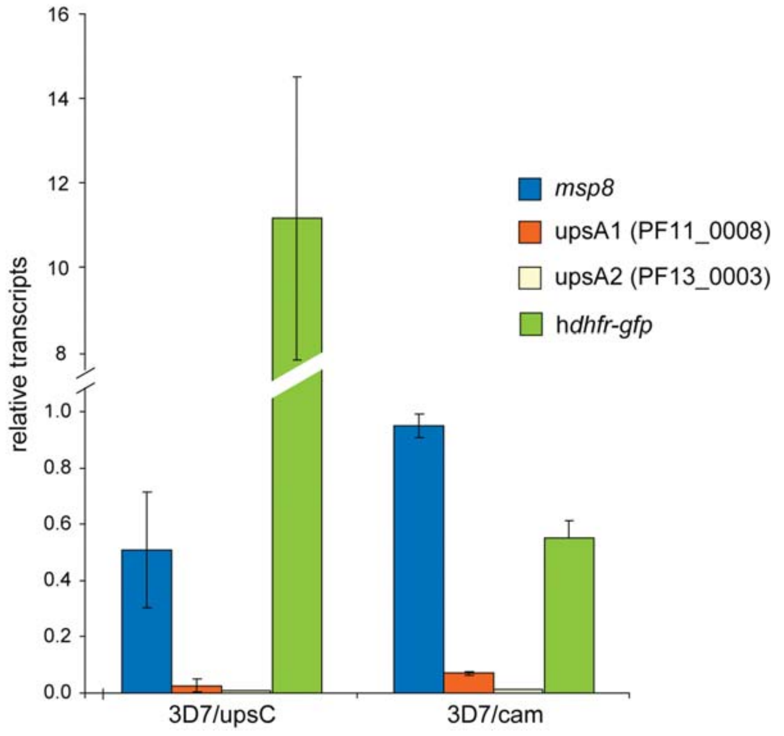

Fig. S5. upsA *var* genes are not up-regulated in WR-selected 3D7/upsC *var* knock-down parasites. Note the 300- to 1000-fold reduced relative transcript abundance of individual upsA *var* genes compared to the *var* promoter-driven h*dhfr-gfp* reporter in 3D7/upsC. Relative transcript levels were determined by qRT-PCR and have been normalised against the house-keeping gene arginyl-tRNA synthetase (PFL0900c). h*dhfr-gfp* transcripts were additionally adjusted for plasmid copy numbers and thus reflect the activity of a single episomal promoter (upsC in 3D7/upsC; *cam* in 3D7/cam). *msp8* is a ring stage-specific control gene. Total RNA was harvested three times independently at TP1 (6-14 hpi). Values represent the mean +/- s.d.

Figure S6

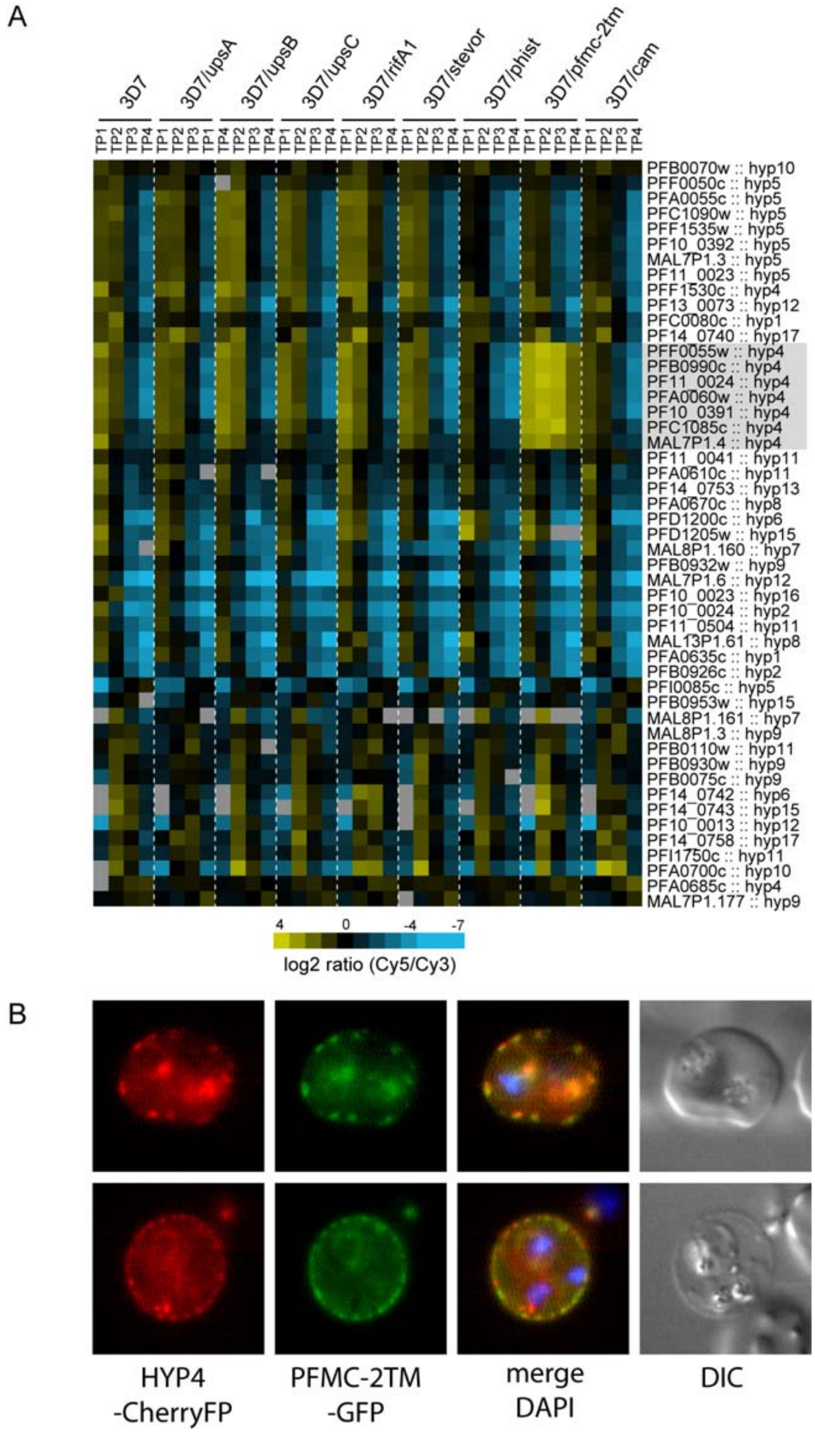
A
3D7
3D7/upsA
3D7/upsB
3D7/upsC
3D7/rifA1
3D7/stevor
3D7/phist
3D7/pfmc-2tm
3D7/cam
TP1
TP2
TP3
TP4
PFB0070w :: hyp10
PFF0050c :: hyp5
PFA0055c :: hyp5
PFC1090w :: hyp5
PFF1535w :: hyp5
PF10_0392 :: hyp5
MAL7P1.3 :: hyp5
PF11_0023 :: hyp5
PFF1530c :: hyp4
PF13_0073 :: hyp12
PFC0080c :: hyp1
PF14_0740 :: hyp17
PFF0055w :: hyp4
PFB0990c :: hyp4
PF11_0024 :: hyp4
PFA0060w :: hyp4
PF10_0391 :: hyp4
PFC1085c :: hyp4
MAL7P1.4 :: hyp4
PF11_0041 :: hyp11
PFA0610c :: hyp11
PF14_0753 :: hyp13
PFA0670c :: hyp8
PFD1200c :: hyp6
PFD1205w :: hyp15
MAL8P1.160 :: hyp7
PFB0932w :: hyp9
MAL7P1.6 :: hyp12
PF10_0023 :: hyp16
PF10_0024 :: hyp2
PF11_0504 :: hyp11
MAL13P1.61 :: hyp8
PFA0635c :: hyp1
PFB0926c :: hyp2
PFI0085c :: hyp5
PFB0953w :: hyp15
MAL8P1.161 :: hyp7
MAL8P1.3 :: hyp9
PFB0110w :: hyp11
PFB0930w :: hyp9
PFB0075c :: hyp9
PF14_0742 :: hyp6
PF14_0743 :: hyp15
PF10_0013 :: hyp12
PF14_0758 :: hyp17
PFI1750c :: hyp11
PFA0700c :: hyp10
PFA0685c :: hyp4
MAL7P1.177 :: hyp9
4
0
-4
-7
log2 ratio (Cy5/Cy3)
B
HYP4
-CherryFP
PFMC-2TM
-GFP
merge
DAPI
DIC

Fig. S6. Up-regulation of *hyp4* transcription in 3D7/pfmc-2tm.

A. Heat map showing relative expression levels (log2 ratios) of all *hyp* family genes in all cell lines and time points (TP1 – TP4). *hyp4* family members up-regulated in 3D7/pfmc-2tm are highlighted in grey.

B. Live cell fluorescence microscopy shows co-localisation of HYP4-CherryFP and PFMC2-TM-GFP at Maurer's clefts in 3D7/HYP4-CherryFP/PFMC-2TM_GFP trophozoites.

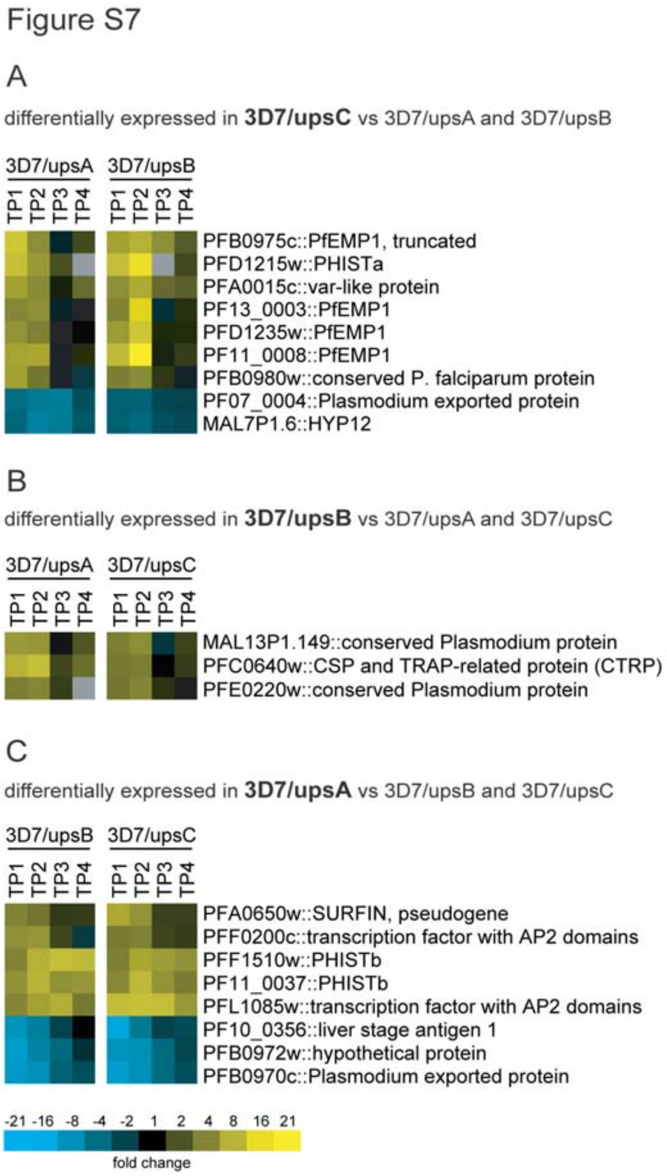

Fig. S7. Differential expression in parasite lines selected for activation of different *var* promoter subtypes. The colour scale indicates fold changes in relative transcript abundance ($2^{\Delta \log2\ ratios}$).

A. Genes with >3-fold changes in relative expression at TP1 and TP2 in 3D7/upsC compared to both 3D7/upsA and 3D7/upsB. Nine genes were differentially expressed in 3D7/upsC, all of which are heterochromatic (Flueck *et al*., 2009) and encode known or predicted exported proteins (Sargeant *et al*., 2006) including one PHISTa paralog (Fig. 6A). Interestingly, a previous study reported up-regulation of a *phista* gene in parasites selected to express an upsC-type PfEMP1 (Mok *et al*., 2007). Further, two

pairs of directly adjacent genes, PFB0975c/PFB0980w and PF07_0004/MAL7P1.6, were similarly up- and down-regulated, respectively, suggesting that local alterations in the heterochromatic environment affected their transcription equally.

B. Genes with >3-fold changes in relative expression at TP1 and TP2 in 3D7/upsB compared to both 3D7/upsA and 3D7/upsC. Two conserved *Plasmodium* proteins with unknown function and *ctrp* (Trottein *et al.*, 1995) were up-regulated in 3D7/upsB (Fig. 6B). Transcription of these three genes is higher in gametocytes compared to intra-erythrocytic stages (Le Roch *et al.*, 2003). Hence, it is possible that these changes were due to a higher proportion of sexual stages in the 3D7/upsB population.

C. Genes with >3-fold changes in relative expression at TP1 and TP2 in 3D7/upsA compared to both 3D7/upsB and 3D7/upsC. Eight genes were differentially expressed in 3D7/upsA, seven of which are associated with PfHP1 (Flueck *et al.*, 2009) (Fig. 6C). Two of the three down-regulated genes, PFB0972w and PFB0970c, fall into the same heterochromatic cluster that was up-regulated in 3D7/upsC (see above). Two up-regulated genes encode PHISTb paralogs and two encode members of the ApiAP2 family of putative transcription factors. One of the *apiap2* genes codes for PfSIP2 (PFF0200c), which has been implicated in upsB silencing (Flueck *et al.*, 2010). The second *apiap2* gene (PFL1085w) is positioned in facultative heterochromatin (Flueck *et al.*, 2009) and thus predisposed to variegated expression.

Table S1: List of primers used in this study

**Primers used for cloning**	**Gene ID**	**primer sequence 5' --> 3'**	**RE sites**
dhfr-Fwd-B-N		agct**ggatccgcggccgc**aaaacaatgcatggttcgctaaactg	BamHI, NotI
dhfr-Rev-S		agcta**gtcgacgcagc**atcattcttctcatatacttcaa	SalI
gfp-Fwd-S		agct**gtcgacgctgct**atgagtaaaggagaagaacttT	SalI
gfp-Rev-A-H		agct**aagcttcctagg**ttaactagtaccggttttgta	AvrII, HindIII
intron-F-M-S		catg**caattg**aaggccttatgtatttatattgaaaaag	MfeI, StuI
intron-R-M-S		catg**caattg**aaggccttgtgtatatgtatgtgtatg	MfeI, StuI
upsA_Fwd	PF13_0003	cagt**agatct**tttctttttttattgtgatacg	BglII
upsA_Rev		cagt**gcggccgctt**taaaaacaatattatttcttcatgg	NotI
upsB_Fwd	PFL0005w	cagt**agatct**cctataggtacgaatatagg	BglII
upsB_Rev		cagt**gcggccgc**gtcatacatatattttggagc	NotI
upsC_Fwd	PFL1960w	cagt**agatct**ttatgttggtacattatacatg	BglII
upsC_Rev		cagt**gcggccgc**ttttgtttttttgtttatcgttcg	NotI
rifA1_Fwd	PF13_0004	cagt**agatct**ttttaaaaacaatattatttcttcatgg	BglII
rifA1_Rev		cagt**gcggccgc**ttttctttttttattgtgatacg	NotI
stevor_Fwd	PFL2610w	tacg**agatct**attaaatcaat**c**aatatatggttcc	BglII
stevor_Rev		cagt**gcggccgc**taattcaaaaaaataatatgttatatttc	NotI
phist_Fwd	PFL2540w	cagt**ggatcc**acttatatttttatgaattgtagg	BamHI
phist_Rev		cagt**gcggccgc**gatttatacatcgtatagatacg	NotI
pfmc-2tm_Fwd		tcga**agatct**gaataaaagaatactatacattaacag	BglII
pfmc-2tm_Rev		cagt**gcggccgc**gtgtattctaatatatagatgtg	NotI
Bsd_F_BglII		cagt**agatct**atggcacctttgtctcaag	BglII
PbDT_R_KpnI		cagt**ggtacc**ggccgcctaccctgaag	KpnI
CherryFP_Fwd		gact**gagctc**atggtgagcaagggcgaggag	SacI
CherryFP_Rev		gact**gtcgac**ttacttgtacagctcgtccatgc	SalI
GFP_Fwd		tgcag**cggccgc**atgagtaaaggagaagaacttttcactg	NotI
GFP_Rev		gtcat**gtcgac**accggttttgtatagttcatccatgcc	SalI
PFMC-2TM Fwd	PFF0060w	caca**ggatcc**atgtttcattatatttataaaatatata	BamHI
PFMC-2TM_Rev		tata**gcggccgc**cttttgttatttgctttttg	NotI
HYP4_Fwd	unknown	atat**ggatcc**atgaactactttctgtcactt	BamHI
HYP4_Rev		tata**gctagc**tttaaatatttttaaatcatgttctg	NheI
**Primers used for qPCR**	**Gene ID**	**primer sequence 5' --> 3'**	**length**
arginyl-tRNA synthetase_Fwd	PFL0900c	aagagatgcatgttggtcattt	117
arginyl-tRNA synthetase_Rev		gagtaccccaatcacctaca	
bsd_Fwd		acagcgtcgccagcgcagctctctcta	151
bsd_Rev		atcgcgacgatacaagtcaggttgccagct	
gfp_Fwd		acacttgtcactactttcgcgtatggtcttc	174
gfp_Rev		accttcaaacttgacttcagcacgtgtcttgtagt	
msp8_Fwd	PFE0120c	tgacgcaaaagcaagggacaacaataataatgatga	156
msp8_Rev		tcatcgtcatcatcattatcatcatcatcatcacc	
upsA1_Fwd	PF11_0008	gcacggctaccacagagacaa	155
upsA1_Rev		cgtcatcatcgtcttcgttt	
upsA2_Fwd	PF13_0003	cacaggtatgggaagcaatg	154
upsA2_Rev		ccatacagccgtgactgttc	

Table S1: List of primer used in this study. qPCR primers used to amplify arginyl-tRNA synthetase (PFL0090c) were modified from Frank *et al.* (Frank *et al.,* 2006). Primers to detect upsA *var* gene transcripts (PF13_0003 and PF11_0008) are from Salanti *et al.* (Salanti *et al.,* 2003).

References

Flueck,C., Bartfai,R., Niederwieser,I., Witmer,K., Alako,B.T., Moes,S. *et al.* (2010) A major role for the *Plasmodium falciparum* ApiAP2 protein PfSIP2 in chromosome end biology. *PLoS Pathog* **6**: e1000784.

Flueck,C., Bartfai,R., Volz,J., Niederwieser,I., Salcedo-Amaya,A.M., Alako,B.T. *et al.* (2009) *Plasmodium falciparum* heterochromatin protein 1 marks genomic loci linked to phenotypic variation of exported virulence factors. *PLoS Pathog* **5**: e1000569.

Frank,M., Dzikowski,R., Costantini,D., Amulic,B., Berdougo,E., and Deitsch,K. (2006) Strict pairing of *var* promoters and introns is required for var gene silencing in the malaria parasite *Plasmodium falciparum*. *J Biol Chem* **281**: 9942-9952.

Le Roch,K.G., Zhou,Y., Blair,P.L., Grainger,M., Moch,J.K., Haynes,J.D. *et al.* (2003) Discovery of gene function by expression profiling of the malaria parasite life cycle. *Science* **301**: 1503-1508.

Mok,B.W., Ribacke,U., Winter,G., Yip,B.H., Tan,C.S., Fernandez,V. *et al.* (2007) Comparative transcriptomal analysis of isogenic *Plasmodium falciparum* clones of distinct antigenic and adhesive phenotypes. *Mol Biochem Parasitol* **151**: 184-192.

Salanti,A., Staalsoe,T., Lavstsen,T., Jensen,A.T., Sowa,M.P., Arnot,D.E. *et al.* (2003) Selective upregulation of a single distinctly structured *var* gene in chondroitin sulphate A-adhering *Plasmodium falciparum* involved in pregnancy-associated malaria. *Mol Microbiol* **49**: 179-191.

Sargeant,T.J., Marti,M., Caler,E., Carlton,J.M., Simpson,K., Speed,T.P., and Cowman,A.F. (2006) Lineage-specific expansion of proteins exported to erythrocytes in malaria parasites. *Genome Biol* **7**: R12.

Struck,N.S., de Souza,D.S., Langer,C., Marti,M., Pearce,J.A., Cowman,A.F., and Gilberger,T.W. (2005) Re-defining the Golgi complex in *Plasmodium falciparum* using the novel Golgi marker PfGRASP. *J Cell Sci* **118**: 5603-5613.

Trottein,F., Triglia,T., and Cowman,A.F. (1995) Molecular cloning of a gene from *Plasmodium falciparum* that codes for a protein sharing motifs found in adhesive molecules from mammals and plasmodia. *Mol Biochem Parasitol* **74**: 129-141.

Voss,T.S., Healer,J., Marty,A.J., Duffy,M.F., Thompson,J.K., Beeson,J.G. *et al.* (2006) A *var* gene promoter controls allelic exclusion of virulence genes in *Plasmodium falciparum* malaria. *Nature* 439: 1004-1008.
